# The GCKIII Kinase Sps1 and the 14-3-3 Isoforms, Bmh1 and Bmh2, Cooperate to Ensure Proper Sporulation in *Saccharomyces cerevisiae*

**DOI:** 10.1371/journal.pone.0113528

**Published:** 2014-11-19

**Authors:** Christian J. Slubowski, Scott M. Paulissen, Linda S. Huang

**Affiliations:** Department of Biology, University of Massachusetts Boston, Boston, Massachusetts, United States of America; Georg-August-University of Göttingen Institute of Microbiology & Genetics, Germany

## Abstract

Sporulation in the budding yeast *Saccharomyces cerevisiae* is a developmental program initiated in response to nutritional deprivation. Sps1, a serine/threonine kinase, is required for sporulation, but relatively little is known about the molecular mechanisms through which it regulates this process. Here we show that *SPS1* encodes a bona-fide member of the GCKIII subfamily of STE20 kinases, both through phylogenetic analysis of the kinase domain and examination of its C-terminal regulatory domain. Within the regulatory domain, we find Sps1 contains an invariant ExxxPG region conserved from plant to human GCKIIIs that we call the EPG motif; we show this EPG motif is important for *SPS1* function. We also find that Sps1 is phosphorylated near its N-terminus on Threonine 12, and that this phosphorylation is required for the efficient production of spores. In Sps1, Threonine 12 lies within a 14-3-3 consensus binding sequence, and we show that the *S. cerevisiae* 14-3-3 proteins Bmh1 and Bmh2 bind Sps1 in a Threonine 12-dependent fashion. This interaction is significant, as *BMH1* and *BMH2* are required during sporulation and genetically interact with *SPS1* in sporulating cells. Finally, we observe that Sps1, Bmh1 and Bmh2 are present in both the nucleus and cytoplasm during sporulation. We identify a nuclear localization sequence in Sps1 at amino acids 411–415, and show that this sequence is necessary and sufficient for nuclear localization. Taken together, these data identify regions within Sps1 critical for its function and indicate that *SPS1* and 14-3-3s act together to promote proper sporulation in *S. cerevisiae*.

## Introduction

Yeast deprived of a fermentable carbon source and nitrogen undergo sporulation [1]. Sporulation begins with meiosis, which results in the production of four haploid nuclei from a single diploid cell. These four nuclei are encapsulated by the prospore membrane, which acts as the template for spore wall deposition. The spore wall differs from the vegetative cell wall, and contains the spore-specific chitosan and dityrosine layers that protect the spores during times of harsh environmental stress. Sporulation is a highly regulated process, and *SPS1*, which encodes a STE20 family serine/threonine kinase, is essential for sporulation [2].

STE20 family kinases are highly conserved from yeast to mammals and are divided into two subgroups, the p21-activated kinases (PAKs) and the germinal center kinases (GCKs) [3], [4]. These two subgroups are distinguished both by the phylogenetic relationships among their kinase domains and by their domain architectures: In PAKs, the kinase domain is C-terminal to the regulatory domain, and this is reversed in GCKs [5]. Within the GCKs, the GCKIII subfamily of kinases includes the mammalian kinases MST3, MST4, and YSK1/SOK1/STK25 [3], which have been implicated in processes such as apoptosis [6] and axon outgrowth [7], and may be involved in diseases such as Alzheimer's [8], type 2 diabetes [9], Parkinson's disease [10], and cerebral cavernous malformations [4].

In *S. cerevisiae, SPS1* is required for proper sporulation. In particular, previous work has shown that *SPS1* is required for the proper localization of the Gsc2, Chs3, and Gas1 enzymes involved in the construction of the spore wall [2], [11], [12]. In addition, Sps1 may play a role in histone modification [13], although whether this role is direct is currently unclear. *SPS1* has also been shown to regulate yeast replicative lifespan [14].

14-3-3 proteins are phosphopeptide binding proteins found in all eukaryotes [15]. There are seven 14-3-3 isoforms in mammals, at least thirteen in plants, and two in yeasts [16]. 14-3-3 family proteins function in a diverse range of biological processes and are implicated in human diseases [17]–[27].

At the molecular level, 14-3-3 proteins are acidic, readily form dimers and bind other proteins using a conserved binding groove [28]. Binding by 14-3-3 proteins has been shown to affect protein function through multiple mechanisms which include acting as a scaffold to facilitate interaction between proteins, modulating protein degradation rate, and altering protein subcellular localization [29]. 14-3-3 binding to substrates in a phosphorylation dependent manner was first shown between 14-3-3ζ and a serine-phosphorylated Raf-1 peptide [30]. Subsequently three different consensus sequences for 14-3-3 binding have been identified: RSX(pS/pT)XP, RXXX(pS/pT)XP [31] and (pS/pTX)(1–2)-COOH [32] (where pS/pT indicates a phosphoserine or phosphothreonine respectively and X represents any amino acid).

The *Saccharomyces cerevisiae* 14-3-3 homologs are encoded by *BMH1* and *BMH2*. Both Bmh1 and Bmh2 are expressed in vegetatively growing cells, although Bmh1 is the major isoform [33]–[35]. In most strain backgrounds, 14-3-3s are essential [36]. However, both *BMH1* and *BMH2* can be removed in the Σ1278b background, a strain in which they have been shown to bind to the kinase, Ste20, and regulate MAPK signaling during pseudohyphal growth [37]. Other 14-3-3 functions in *S. cerevisiae* include: cell cycle regulation [38], DNA replication [39], TOR-signaling [40], PKA signaling [41], transcription [42], cation homeostasis [43], Golgi function [44], lifespan regulation [45], rapamycin-mediated transcription [46], and the spindle position checkpoint [47].

In this study, we use phylogenetic analysis to determine the relationship of Sps1 to other Ste20 kinases, and demonstrate that Sps1 is a bona-fide member of the GCKIII family of STE20 kinases. Our comparative analyses also identify a C-terminal region in GCKIII kinases that is conserved from yeast to mammal to plant, and we show that this region is important for Sps1 function. To obtain insight into the regulatory interactions of Sps1, we map phosphorylation sites on Sps1 and identify threonine 12 (T12) as a residue important for Sps1 function and efficient sporulation. We show that Sps1-T12 is required for the physical interaction between Sps1 and the 14-3-3 proteins Bmh1 and Bmh2. We describe a role for 14-3-3 proteins in sporulation, and demonstrate that the relative levels of Bmh1 and Bmh2 change during sporulation. We show that Sps1 and 14-3-3 proteins are present in both the nucleus and cytoplasm during sporulation, and we identify a nuclear localization signal for Sps1. Because we see both a physical and genetic interaction between 14-3-3 proteins and Sps1, we propose that Bmh1, Bmh2, and Sps1 act together during sporulation to regulate spore formation.

## Materials and Methods

### Plasmids used in this study

All plasmids used in this study can be found in Table S1 and all primers in Table S2. Construction details are described below. All plasmid inserts amplified using PCR were verified by sequencing.

pCS22 (pRS426-P_TEF2_-*GFP-SPS1*) was constructed by amplifying the *SPS1* coding sequence from genomic SK1 DNA using primers OLH1128 and OLH1129 and then cutting both the amplified DNA and pRS426-P_TEF2_-*GFP-SPO71(1–1245)* [48] with HindIII and XhoI restriction enzymes. The *SPS1* ORF was then ligated into the GFP containing plasmid so that GFP was N-terminally fused to *SPS1*.

pCS20 (pRS426-P_TEF2_-*SBP-SPS1*) was created by amplifying SBP (Streptavidin Binding Peptide) from plasmid pMK33-CTAP(SG) [49] using primers OLH1132 and OLH1133. The PCR product as well as plasmid pCS22 (pRS426-P_TEF2_-*GFP-SPS1*) were cut with restriction enzymes EcoRI and HindIII. This allowed ligation of SBP in place of GFP.

pCS65 (pRS426-P_TEF2_ *GFP-sps1-ggaga*) and pCS130 (pRS426-P_TEF2_ *GFP-sps1-arappa*) were constructed by site-directed mutagenesis of pCS20 (pRS426-P_TEF2_-*SBP-SPS1*) using primers OLH1182/OLH1183 and OLH1362/OLH1363 respectively. The mutagenized ORFs were then cloned into pCS22 (pRS426-P_TEF2_-*GFP-SPS1*) in place of *SPS1* using the HindIII and XhoI restriction sites.

pCS28 (pRS426-P_TEF2_-*SBP-sps1-K47R*) was constructed by site-directed mutagenesis of pCS22 (pRS426-P_TEF2_-*GFP-SPS1*) and subsequent cloning into pCS20 (pRS426-P_TEF2_-*SBP-SPS1*) using restriction sites HindIII and XhoI.

pCS75 (pRS426-P_TEF2_-*GFP-GST-URA3*) was constructed by first amplifying GST out of plasmid pGEX-4T-3 using the primer combination OLH1258/OLH1259, cutting both the PCR product (which lacked 9 amino acids from the C-terminal due to an endogenous XhoI site) and pCS22 (pRS426-P_TEF2_-*GFP-SPS1*) with HindIII and XhoI.

pCS60 (pRS426-P_TEF2_-*GFP-GST-SPS1(387–438)*) and pCS78 (pRS426-P_TEF2_-*GFP-GST-sps1-ggaga-(387–438)*) were constructed in the same manner as pCS75 (pRS426-P_TEF2_-*GFP-GST-URA3*) except the primer combination OLH1258/OLH1260 was used to generate a GST product without a stop codon. OLH1261 and OLH1262 were then used to amplify the *SPS1* NLS region from pCS22 (pRS426-P_TEF2_-*GFP-SPS1*) and pCS65 (pRS426-P_TEF2_-*GFP-sps1-ggaga*) respectively. Overlap PCR was performed using the PCR products generated in the above reactions with primers OLH1258 and OLH1262. Overlap PCR resulted in products: *GST-SPS1(387–438)* and *GST-sps1-ggaga-(387–438)* respectively. These products, as well as pCS22 (pRS426-P_TEF2_-*GFP-SPS1*), were then cut with the restriction enzymes HindIII and XhoI and ligated into pCS22 (pRS426-P_TEF2_-*GFP-SPS1*).

pCS96 (pRS316-P_TEF2_-*SBP-SPS1*) was created by cutting pCS20 (pRS426-P_TEF2_-*SBP-SPS1*) with SacI and KpnI and ligating the resulting product into the identically cut pRS316.

pCS107 (pRS316-P_TEF2_-*SBP*) was constructed by first amplifying SBP from pMK33-CTAP(SG) using the primer combination OLH1132/OLH1226 and then cutting the resulting product, as well as pCS96 (pRS316-P_TEF2_-*SBP-SPS1*), with EcoRI and XhoI followed by ligation.

pCS98 (pRS316-P_TEF2_-*SBP-sps1-T12A*) was constructed by mutagenic PCR of pCS20 (pRS426-P_TEF2_-*SBP-SPS1*) that changed the codon for T12 using primers OLH1281 and OLH1282. The mutagenized *sps1* ORF was then excised using HindIII and XhoI restriction sites and ligated into pCS96 (pRS316-P_TEF2_-*SBP-SPS1*).

pCS99 (pRS316-P_SPS1_-*SBP-SPS1*) was created by first amplifying the promoter region of *SPS1* using the primer combination OLH1230/OLH1257. The PCR product and pCS96 (pRS316-P_TEF2_-*SBP-SPS1*) were cut with SacI and EcoRI and the *SPS1* promoter was ligated in place of the *TEF2* promoter.

pCS100 (pRS316-P_SPS1_-*SBP-sps1-T12A*) was created by cutting *sps1-T12A* out of pCS98 (pRS316-P_TEF2_-*SBP-sps1-T12A*) using HindIII and XhoI and ligating it in place of *SPS1* in pCS99 (pRS316-P_SPS1_-*SBP-SPS1*), which was also cut with the same enzymes.

pCS145 (pRS316-P_SPS1_-*sfGFP-sps1-T12A*) was constructed by first PCR amplifying sfGFP from pDHL1029 using primers OLH1416 and OLH1417 and then cutting the resulting PCR product as well as pCS100 (pRS316-P_SPS1_-*SBP-sps1-T12A*) with EcoRI and HindIII. sfGFP was then ligated in place of SBP.

pCS146 (pRS316-P_SPS1_-*sfGFP-SPS1*) was constructed by cutting pCS99 (pRS316-P_SPS1_-*SBP-SPS1*) and pCS145 (pRS316-P_SPS1_-*sfGFP-sps1-T12A*) with the restriction enzymes HindIII and XhoI and ligating *SPS1* in place of *sps1-T12A*.

pCS47 (pBSIIKS+: *_−137_ SPS1_+297_*) was constructed by amplifying genomic DNA using primers OLH778 and OLH1195. The resulting product, as well as pBSIIKS+, were cut with the restriction enzymes ClaI and SpeI followed by ligation.

pCS159 was constructed by PCR mutagenesis of pCS47 (pBSIIKS+: *_−137_ SPS1_+297_*) using primers OLH1466 and OLH1467.

### Strains used in this study

All strains in this study were derived from the SK1 background [50] (list of strains, Table S3). C-terminal tagging, gene disruptions, and gene deletions were accomplished by PCR mediated recombination [51], [52]. All of the above genome changes were confirmed by diagnostic PCR.

The *dit1::TRP1* and *bmh1::TRP1* alleles were created using primers OLH608/OLH609 and OLH1309/OLH1328, respectively, in conjunction with pCgW.

The *sps1::LEU2* and *sps1::HIS3* alleles were created using primers OLH131/OLH132 in conjunction with pLEU2 and pHIS3, respectively. The *SPS1-zz-URA3* and *sps1ΔEPG-zz-URA3* alleles were created using primers OLH391/OLH392 and OLH487/OLH488, respectively, with pBS1365. The *SPS1-13xmyc-TRP1* allele was created using primers OLH389/OLH390 in conjunction with pFA6a-13Myc-TRP1. The *BMH1-GFP-TRP1* and *BMH2-GFP-TRP1* alleles were created using primers OLH1305/OLH1327 and OLH1276/OLH1277, respectively, along with pFA6a-GFP(S65T)-TRP1. The *bmh2::URA3*, *URA3-SPS1* and *SBP-sps1(S345::URA3)* alleles were created using primers OLH1311/OLH1312, OLH826/OLH827 and OLH1459/OLH1460, respectively, in conjunction with pURA3. The DNA product from OLH1459/OLH1460 and pURA3 was then transformed into a strain carrying the *SBP-SPS1* allele (described below).

The alleles: *SBP-SPS1*, *SBP-sps1-T12A*, *sfGFP-SPS1*, and *sfGFP-sps1-T12A* were created by replacement of the *URA3* in the *URA3-SPS1* allele (described above) by PCR amplified DNA specific to each allele (see below). Counter-selection on plates containing 5-fluoroorotic acid (5FOA; Zymo Research) was used to screen for successful replacement of *URA3* as previously described [50]. The primers OLH780/OLH513 were used with pCS99, pCS100, pCS146 and pCS145 to amplify DNA for the creation of the alleles: *SBP-SPS1*, *SBP-sps1-T12A*, *sfGFP-SPS1*, and *sfGFP-sps1-T12A*, respectively. The allele *SBP-sps1-S345A* was created by 5FOA counter-selection as described above using the *SBP-sps1(S345::URA3)* allele and DNA generated using primers OLH507/OLH796 and pCS159. All PCR mediated alleles were verified afterward by DNA sequencing.

### Fluorescence microscopy

Microscopy was done using a 100× (NA 1.45) objective on a Zeiss Axioskop Mot2. Images were taken using an Orca-ER cooled charge-coupled device camera (Hamamatsu) using Openlab 4.04 (Perkin Elmer) software. All imaging was done using live cells.

### Yeast growth conditions

Yeast cells were induced to sporulate as described previously [50]. In brief, yeast cells were grown to saturation in YPD (2% Peptone, 1% Yeast Extract, 2% Dextrose), and transferred to pre-sporulation media YPA (2% Peptone, 1% Yeast Extract, 1% Potassium Acetate). Cells were grown in pre-sporulation media overnight, and then shifted to sporulation media (2% Potassium Acetate). Cells grown in log phase were grown in either YPD or the appropriate selective medium to approximately OD_600_ 0.8.

### Scoring of sporulation phenotypes

Spore efficiency was measured using liquid sporulation cultures from a minimum of three biological replicates. Meiotic progression was determined by examining Htb2-mCherry [48]. Cultures were allowed to sporulate in liquid media for 24 hours. Yeast cells were scored for sporulation if at least one refractile spore was formed; a minimum of 200 cells was counted for each culture.

To determine the number of spores formed in each ascus, three biological replicates were tested for each strain. Cultures were sporulated in liquid sporulation media for 24 hours. Yeast cells were scored for number of refractile spores formed per ascus. Cells that did not form refractile spores were not counted.

The dityrosine fluorescence assay was performed as described [53]. In short, cells were grown on YPD plates for 24 hours then transferred to SPO plates with a nitrocellulose filter, incubated for another 24 hours and then exposed to UV light. The nitrocellulose membrane was then imaged using a digital camera.

Spore viability was assayed by the dissection of 25 tetrads per strain [54]. Dissected spores were allowed to grow on YPD plates for 48 hours.

Spore wall permeability assays were carried out as previously described [55]. In brief, yeast strains were transformed with pRS424-ssGFP, induced to sporulate, and visualized for GFP in the ascal cytoplasm or trapped within the extracellular space between the plasma membrane and the spore wall. For each strain 100 cells were counted from three biological replicates for a total of 300 total cells counted per strain. Only cells with refractile spores were counted.

### Protein immunoblotting

Protein lysates for immunoblotting were prepared using trichloroacetic acid (TCA) denaturation, as described [31]. After TCA precipitation, proteins were re-suspended in SDS-PAGE sample buffer [50] boiled for 5 minutes and separated by SDS-PAGE. Proteins were transferred onto polyvinylidene fluoride (GE Healthcare) and probed with the following antibodies: rabbit pre-immune anti-sera (gift from K. Benjamin) at 1∶1000 to detect the zz epitope (which contains two copies of the z-domain of Protein A [52]); mouse monoclonal antibody 9E10 (Covance) at 1∶1000 to detect the Myc epitope; mouse monoclonal JL-8 (BD Living Colors) at 1∶1000 to detect GFP, mouse monoclonal SB19-C4 (Santa Cruz Biotechnology) at 1∶1000 to detect SBP, mouse monoclonal 22C5D8 (abcam) at 1∶1000 to detect Pgk1, rabbit polyclonal anti-Bmh [35] at 1∶10000 to detect Bmh1 and Bmh2 and rabbit anti-Ndt80 [56] at 1∶1000 to detect Ndt80.

Blots visualized on the Kodak Image Station 4000R with Kodak Molecular Imaging Software v4.0.4 were stained with the appropriate anti-rabbit or anti-mouse HRP conjugated secondary antibodies at 1∶10000 (Jackson ImmunoResearch). Secondary antibodies detected using Supersignal West Dura Extended Duration Substrate (Pierce).

Blots visualized on the Odyssey CLx Infrared Imaging System (LI-COR Biosciences) were blocked using Odyssey blocking buffer (LI-COR) and Goat Anti-Mouse IR Dye 800 CW (LI-COR) at 1∶10000 as a secondary antibody. Protein bands were quantified using Image Studio v3.1 (LI-COR).

### Immunoprecipitation, phosphatase assay and mass spectrometry

Lysates for immunoprecipitation were prepared from 60 OD_600_ of cells, lysed in a Mini Bead Beater8 (Biospec) at 4°C with glass beads in IP buffer (329.2 mM NaCl, 0.0823 mM EDTA, 16.46 mM HEPES, 1.2345 mM MgCl_2_, 0.823% Nonidet P-40) and a protease and phosphatase inhibitor cocktail as described [56]. Sps1-zz was immunoprecipitated using IgG Sepharose (GE Healthcare) and SBP-Sps1 was immunoprecipitated using Streptavidin Plus Ultralink Resin (Pierce).

Sps1-zz immune complexes were washed 4 times in IP buffer and then re-suspended in SDS-PAGE sample buffer, boiled for 5 minutes and separated by SDS- PAGE. SBP-Sps1 immune complexes were washed 4 times in IP buffer and then SBP-Sps1 was eluted using biotin solution (2 mM biotin (Sigma), 0.28% ammonium hydroxide (Sigma)). Eluate was then TCA prepped as described above. The TCA precipitated protein was then resuspended in SDS-PAGE sample buffer, boiled for 5 minutes, and separated by SDS-PAGE.

Phosphatase assays were performed as described [56]. Briefly, Sps1-zz was immunoprecipitated, washed 4 times in IP buffer and then once in pre-phosphatase buffer (50 mM Tris-HCl at pH 7.5, 0.1 mM EDTA, 2 mM MnCl2, 0.1 mg/mL BSA) re-suspended in 50 µL of reaction buffer (50 mM Tris-HCl at pH 7.5, 0.1 mM EDTA, 5 mM DTT, 2 mM MnCl2, 0.01% Brij35, 50 U of λ protein phosphatase (New England Biolabs)). Control reactions omitted phosphatase or added phosphatase inhibitors (50 mM NaF, 50 mM β-glycerophosphate, 2 mM sodium orthovanadate). Reactions were incubated for 30 minutes at 30°C. Beads were then resuspended in SDS-PAGE sample buffer, boiled for 5 minutes and separated by SDS-PAGE.

Samples for mass spectrometry phosphorylation analysis were immunoprecipitated and then resuspended in SDS-PAGE sample buffer, boiled for 5 minutes and separated by SDS-PAGE. Gel bands were excised and sent to the Taplin Mass Spectrometry Facility (Harvard Medical School). In brief, samples underwent a modified in-gel trypsin digestion [57], followed by peptide extraction procedures, separation on a reverse-phase HPLC capillary column [58], electrospray ionization and peptides then entered into an LTQ-Orbitrap mass spectrometer (Thermo Fisher, San Jose, CA). Peptide sequences were determined by matching protein or translated nucleotide databases with the acquired fragmentation pattern by the software program, Sequest [59]. To identify phosphorylated residues, the modification of 79.9663 mass units to serine, threonine, and tyrosine was included in the database searches to determine phosphopeptides and each phosphopeptide that was identified by the Sequest program was also inspected manually.

### Bioinformatics

ScanSite, http://scansite.mit.edu/ [60], was used to predict the 14-3-3 binding site in Sps1. ScanSite was run using the high stringency setting.

For phylogenetic analysis, kinase domains were identified based on PFAM kinase domain (PF00069) and aligned using MUSCLE 3.7 [61]. Multiple sequence alignments were then used for maximum likelihood phylogeny, performed using PhyML v3.0, [62] with LG substitution matrix [63] and Shimodaira-Hasegawa-like (SH-like) approximate likelihood ratio test (aLRT) branch supports [64]. Phylogenetic trees were displayed using FigTree (http://tree.bio.ed.ac.uk/software/).

## Results

### Sps1 is a GCKIII family member

Sps1 has been considered an outlier among Ste20 kinases, as initial analysis of the evolutionary relationships among Ste20 family kinases suggested it belonged to neither GCK nor PAK sub-families [5]. However, we noticed that when compared to human Ste20 kinases, the kinase domain of Sps1 shares ∼50% amino acid identity with the GCKs (e.g., 52% identity with the human GCKIII Ysk1), but only ∼40% with the PAKs (e.g., 40% with the human Pak1). This raised the possibility that rather than being an outlier among Ste20 kinases, Sps1 might belong to the GCK subfamily, and prompted us to revisit the evolutionary relationship of the Sps1 kinase domain to other Ste20 family members.

**Figure 1:**
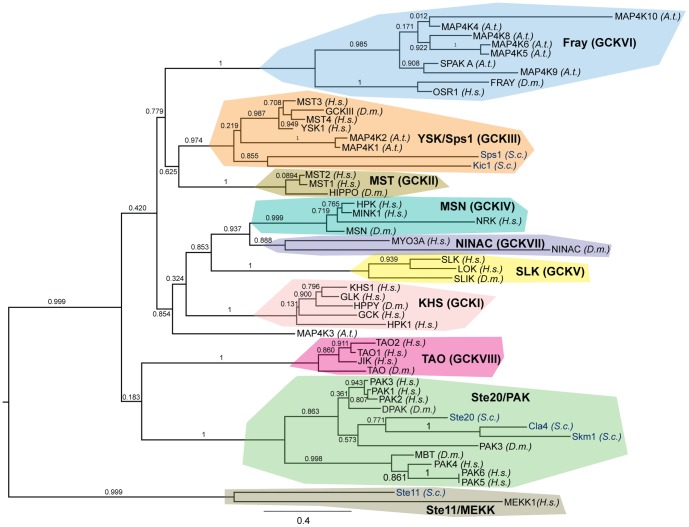
Sps1 is a GCKIII family member. Phylogenetic analysis using maximum likelihood-based methods. Branch support Ps are noted where relevant. (*A.t.: Arabidopsis thaliana*, *D.m.: Drosophila melanogaster*, *H.s.*: *Homo sapiens*, *S.c.: Saccharomyces cerevisiae*.) The GCK subfamilies, the PAK subfamily, and the MEKK outgroup are highlighted in different colors. The *S. cerevisiae* protein names are in blue; all other kinase names are in black.

Phylogenetic analysis of Sps1 was performed to examine relationships among the kinase domain sequences of all five *S. cerevisiae* Ste20 family members (Cla4, Kic1, Skm1, Sps1, and Ste20), as well as all human, *Drosophila melanogaster* and *Arabidopsis thaliana* Ste20 family members. Two MAP Kinase Kinases (MEKKs), *S. cerevisiae* Ste11 and human MEKK1, were used to root the tree, as MEKK and Ste20 kinases form related but distinct gene families (Figure 1). Maximum likelihood-based phylogenetic analysis found, with strong branch support, that Sps1 resides in a monophyletic clade with human Mst3, Mst4, and Ysk1, as well as *Drosophila* GCK-III. As Mst3, Mst4, Ysk1, and GCK-III are canonical GCKIII family members [3]–[5], these data indicate that Sps1 is not an outlier among Ste20 kinases. Our analysis shows that Sps1 is a GCK, and specifically, a member of the GCKIII subfamily. Phylogenetic analysis performed using a different reconstruction approach (the less computationally intense Neighbor Joining method) also indicated Sps1 belongs to the GCKIII family. Consistent with this view, the Sps1 domain architecture also resembles GCK architecture, with the kinase domain located at the amino-terminus. Interestingly, two *Arabidopsis* MAP4Ks also fall within the GCKIII clade, suggesting that the GCKIII kinase lineage diverged from other Ste20 kinases prior to the separation of the plant and opisthokont (yeast/animal) lineages ∼1 billion years ago. The other yeast Ste20 family member with a GCK-like domain architecture, Kic1, is also a member of the GCKIII clade.

**Figure 2:**
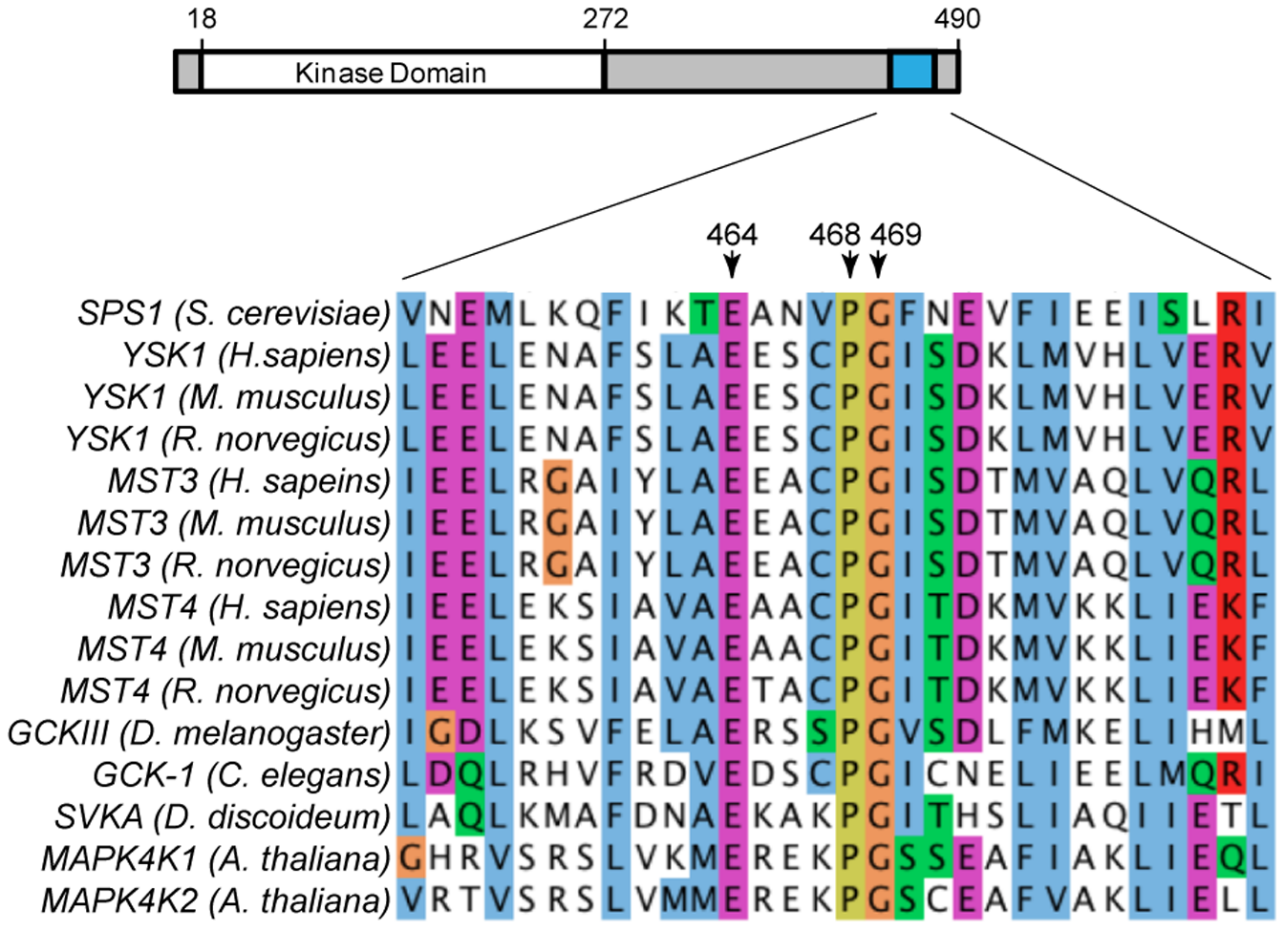
The C-terminal region of Sps1 contains a conserved EPG motif. Alignment of Sps1 residues 453–482 with the relevant regions from the human, mouse, rat, fly, nematode, slime mold, and mustard weed homologs. Amino acids with similar properties are colored using the ClustalX default color scheme [86]: magenta = acidic, red = basic, blue = hydrophobic, green = hydrophilic, orange = glycine, yellow = proline.

As a member of the GCKIII family, Sps1 could share amino acid similarities outside the kinase domain. We performed sequence alignments with Sps1 and other GCKIIIs and found Sps1 contains a conserved region at its C-terminus (Figure 2). This region extends from amino acid 453 to 482 in Sps1, and it includes three amino acids conserved between Sps1 and the animal and plant GCKIIIs (Figure 2). In Sps1, these residues are glutamic acid (E) 464, proline (P) 468, and glycine (G) 469. We call this invariant ExxxPG region the EPG motif. Taken together, the phylogenetic evidence, domain architecture, and C-terminal sequence similarity all support the identification of Sps1 as a member of the GCKIII subfamily of Ste20 kinases.

### The C-terminal EPG motif containing region is required for Sps1 function

To test whether the C-terminal EPG motif containing region is required for Sps1 function, we created the *sps1ΔEPG-zz* allele by removing the last 38 amino acids (and thus deleting the ExxxPG region). The deletion starts from the conserved valine (V) 453 to the C-terminus; this sequence is replaced with the *zz* (two tandem *z* domains from Protein A) epitope [52]. We compared a strain carrying the *sps1ΔEPG-zz* allele to strains carrying the alleles: *SPS1-zz*, *sps1::LEU2* as well as wild type for their ability to form refractile spores (summarized in Table 1). We found that wild type and *SPS1*-zz cells sporulate at 80.7% and 81.7% respectively, while *sps1ΔEPG-zz* reduces sporulation similar to the *sps1* null allele (6.8% and 4.0%, respectively) (Student's *t* test comparison of LH960 and LH961 gives a *P*<0.01). This reduction in sporulation is not due to a reduction in protein level, as the *sps1ΔEPG-zz* mutation does not grossly disrupt steady state levels of Sps1 protein (Figure S1A).

**Table 1:** The EPG motif containing region is required for *SPS1* function.

strain name	relevant genotype	% spores (mean ± S.D.)
LH902	wild type	80.7±2.1
LH960	*SPS1-zz*	81.7±2.8
LH966	*sps1::LEU2*	4.0±1.8
LH961	*sps1ΔEPG-zz*	6.8±2.9

200 cells/culture and a minimum of 3 cultures were tested for each strain.

### Sps1 is a phosphoprotein

**Figure 3:**
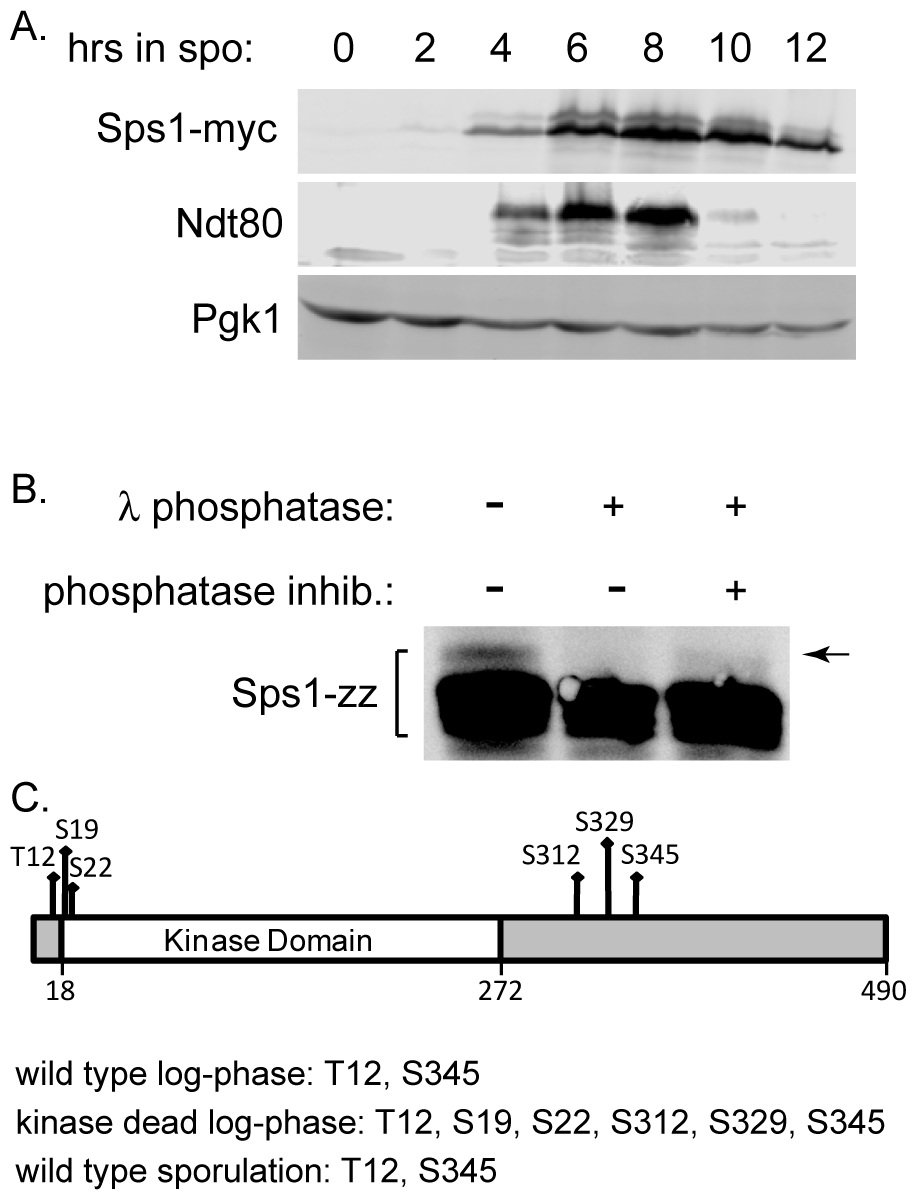
Sps1 is a phosphoprotein. (A) Lysates from LH875 (*SPS1-myc*) were collected throughout sporulation, run on an immunoblot and probed with anti-Myc antibody. Ndt80 was detected using an anti-Ndt80 antibody and used to monitor progression through sporulation [56]. Pgk1 was used as a loading control. (B) Sps1 was immunoprecipitated from lysates collected from LH791 (*SPS1-zz*) harvested 8 hrs into sporulation. The immunoprecipitate was divided and treated with (+) or without (−) λ phosphatase and with (+) or without (−) phosphatase inhibitors. Reactions were run on an immunoblot and then probed with rabbit pre-immune anti-sera, to detect the zz epitope. (C) Mass spectrometry phosphorylation analysis of Sps1 identified several phosphorylation sites. For log-phase cultures, LH988 (containing *SPS1* [pRS426-*SBP-Sps1*]) and LH989 (containing kinase dead *sps1* [pRS426-*SBP-sps1-K47R*]) were grown in selective media and collected during logarithmic phase growth (0.5–0.8 OD_600_). For sporulating cultures, LH954 (*SBP-SPS1*) cells were collected 8 hours into sporulation. Sps1 was immunoprecipitated using the SBP epitope and analyzed by mass spectrometry to identify phosphorylation sites.

We examined Sps1 during sporulation and saw that the level of Sps1 is induced during sporulation and peaks around 6 to 8 hours (Figure 3A), when cells are completing meiosis and starting spore morphogenesis. We see that Sps1 runs as a doublet, suggesting that Sps1 is post-translationally modified.

To test whether this post-translational modification was due to phosphorylation, we carried out a phosphatase assay by examining Sps1 purified from cells 8 hours into sporulation (Figure 3B). When λ phosphatase was added to the immunoprecipitated Sps1 we see that the more slowly migrating band is no longer detectable. When λ phosphatase and phosphatase inhibitors were added together, the higher mobility band can be seen more easily than in the phosphatase only treated sample (Figure 3B). These results indicate that Sps1 is phosphorylated (Figures 3A and 3B). Differences in the intensity of the more slowly migrating phosphorylated band in the phosphatase treatment experiment (Figure 3B) compared to the examination of cell lysates (Figure 3A) may be due to the use of different sample preparations (more native conditions for the phosphatase assay in Figure 3B compared to denaturing conditions for the cell lysates in Figure 3A) or the different epitope tags used in the two different experiments.

To identify potential phosphorylation sites on Sps1, we used mass spectrometric analysis on Sps1 purified from vegetatively growing log-phase cells that expressed Sps1 from a multi-copy plasmid under the *TEF2* promoter and from sporulating cells. Kinase dead *sps1* was also purified from log-phase cells and subjected to mass spectrometric analysis. We found that Sps1 was phosphorylated on threonine (T) 12 and serine (S) 345 in all three experiments. In addition to T12 and S345, four novel phosphorylation sites were found only when the kinase dead version of Sps1 was purified: S19, S22, S312, and S329 (Figure 3C). Thus, Sps1 is phosphorylated on multiple serine and threonine residues, and the phosphorylation of T12 and S345 are not simply due to autophosphorylation by Sps1. Whether S19, S22, S312, or S329 are biologically relevant is unclear, given that we only see these phosphorylation sites on the kinase dead version of Sps1 that is ectopically expressed in log phase cells.

### *sps1-T12A* displays reduced sporulation efficiency

To test the importance of Sps1 T12 or S345 phosphorylation during sporulation, we replaced the *SPS1* locus with either *SBP-sps1-T12A* or *SBP-sps1-S345A*. We did not see any sporulation defect with the *SBP-sps1-S345A* mutant and did not study the effects of this site further. In contrast, the *SBP-sps1-T12A* did affect spore formation. We found that the *sps1* null forms spores 4.0% of the time, while wild type and the *SBP-SPS1* strain sporulate at 80.7% and 85.8% of the time respectively. *SBP-sps1-T12A* forms spores 65.7% of the time, a statistically significant decrease in sporulation efficiency compared to wild type (*P*<0.01; *t* test), although not as pronounced as in an *sps1* null (Table 2).

**Table 2:** *sps1-T12A* has a sporulation defect.

strain name	relevant genotype	% spores (mean ± S.D.)
LH902	wild type	80.7±2.1
LH962	*SBP-SPS1*	85.8±5.0
LH967	*SBP-sps1-S345A*	83.7±2.7
LH968	*SBP-sps1-T12A*	65.7±3.3
LH969	*SBP-sps1-T12A/+*	82.8±3.8

200 cells/culture and a minimum of 3 cultures were tested for each strain.

To examine whether the T12A mutation disrupted Sps1 protein levels or altered its temporal expression profile, we compared wild type SBP-Sps1 and SBP-Sps1-T12A expression during sporulation and found comparable protein levels and similar temporal expression patterns throughout sporulation (Figure S1B).

### *sps1-T12A* is a reduction-of-function mutation defective in spore packaging

Because the *SBP-sps1-T12A* allele did not have a phenotype as severe as the null allele, we wanted to determine the manner in which is disrupts *SPS1* function. First, we tested whether *SPB-sps1-T12A* acts as a dominant negative mutant by creating a heterozygous strain. If *sps1-T12A* were acting as a dominant negative mutation, we would expect a reduction in spore efficiency in heterozygous cells compared to wild type cells. However, we found that there was no reduction in spore formation between wild type (80.7%) and *SBP-sps1-T12A/+* (82.8%; P = 0.4, Student's *t* test).

To test whether *SBP-sps1-T12A* acts as a reduction-of-function allele, we varied the dosage of *sps1*. Consistent with *sps1-T12A* acting as a partial loss-of-function allele, the spore efficiency of *SBP-sps1-T12A/sps1Δ* was reduced compared to *SBP-sps1-T12A/SBP-sps1-T12A* (53.0% compared to 65.67%; P<0.05; *t* test).

To determine whether the spores that are produced in *sps1-T12A* allele show defects, we examined the number of spores formed per ascus when at least one refractile spore was formed in an ascus (Figure S2A), the ability of the outer spore wall layer (the dityrosine layer) to fluoresce [53] (Figure S2B), as well as spore wall permeability using a signal sequence fused to GFP (pRS424-ssGFP; [55]) (Figure S2C). We saw no differences in the spores formed by cells homozygous for the *sps1-T12A* allele compared to wild type cells. We also examined the germination efficiency in *SBP-sps1-T12A* cells by dissecting 25 tetrads each of *SBP-sps1-T12A* and *SBP-SPS1* (only cells that made four refractile tetrads in an ascus were dissected) and did not see any differences in the ability of haploid spores to germinate. Of 100 spores dissected, 98 germinated in the *SPB-SPS1* containing strain, while 96 germinated in the *SBP-sps1-T12A* containing strain. Taken together, these results suggest that while *sps1-T12A* reduces the efficiency of spore packaging, the spores that are packaged and form refractile structures show no obvious defects.

### Sps1 has a consensus 14-3-3 binding site and can physically interact with Bmh1 and Bmh2

**Figure 4:**
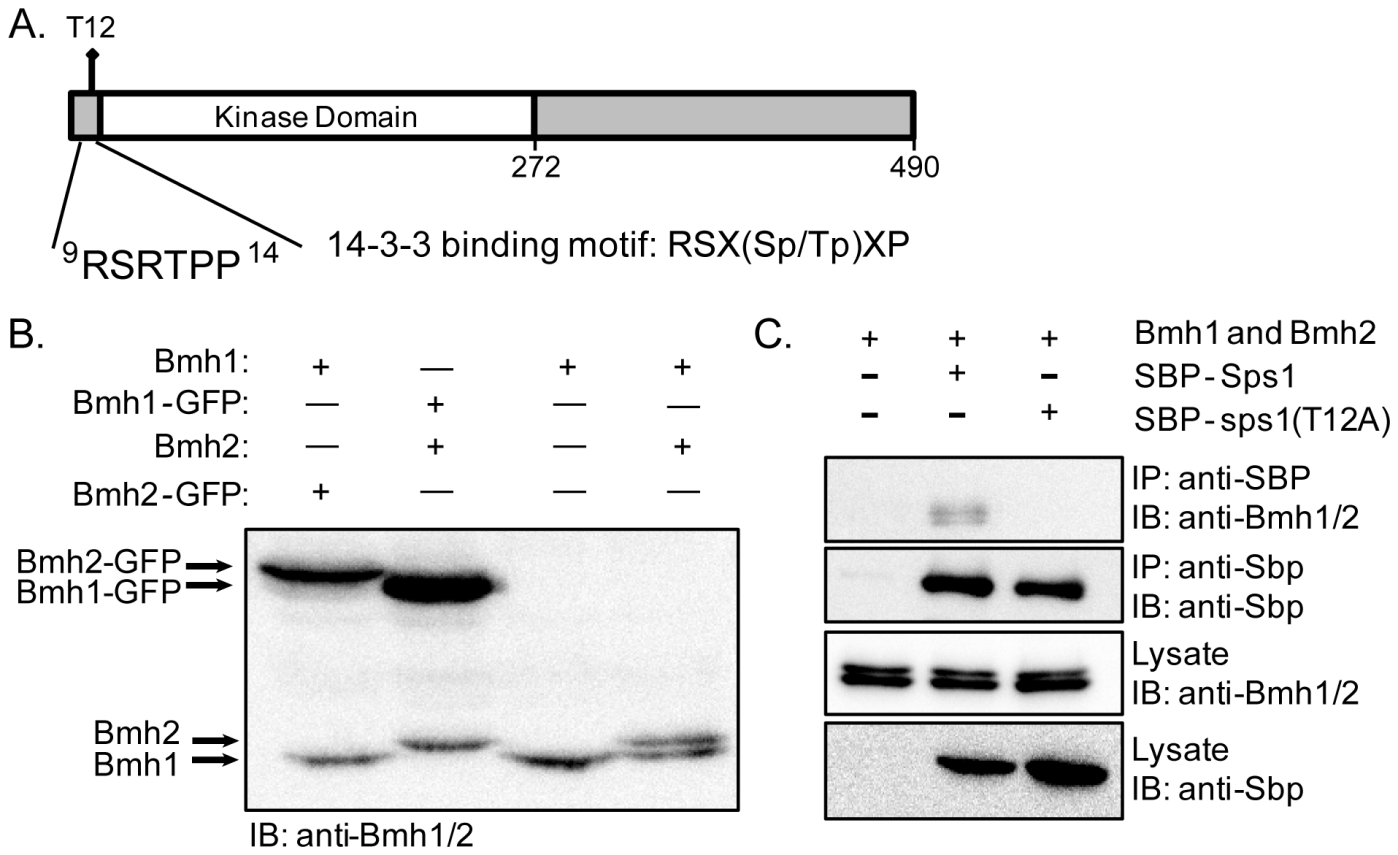
Sps1 can physically interact with Bmh1 and Bmh2. (A) The 14-3-3 consensus sequence within Sps1. (B) Immunoblot probed with anti-Bmh antibody; strains from left to right: LH958 (*BMH2-GFP*), LH957 (*BMH1-GFP*), LH959 (*bmh2Δ*) and LH177 (*WT*) (C) Co-immunoprecipitation between Sps1 and the 14-3-3 homologs, Bmh1 and Bmh2. LH994 (pRS426-*SBP*), LH993 (pRS426-*SBP-SPS1*) and LH992 (pRS426-*SBP-sps1-T12A*), were grown in selective media and harvested during log-phase growth. SBP immunoprecipitation (IP) was performed, and samples were analyzed by probing immunoblots (IB) with antibodies as indicated.

When we examined the region surrounding T12 within the Sps1 protein, we found that this threonine resides within a potential 14-3-3 phosphopeptide binding consensus sequence. The sequence of Sps1 starting from arginine (R) 9 to proline (P) 14 closely matches the 14-3-3 consensus sequence: R-S-X-(pS/pT)-X-P [31] (Figure 4A).

To examine Bmh1 expression in the SK1 strain, we used an anti-Bmh antibody [35]. To test the specificity of the anti-Bmh antibody in the SK1 strain, we examined its ability to recognize Bmh1 and Bmh2 using wild type, *bmh2Δ*, *BMH1-GFP*, and *BMH2-GFP* containing strains, which allowed us to distinguish between the two 14-3-3 isoforms, as appending GFP at the C-terminus of the protein shifts the migration of an isoform (Figure 4B). We found that this antibody can detect both isoforms of 14-3-3 proteins in our strain background, with the more slowly migrating isoform corresponding to Bmh2 while the faster migrating isoform is Bmh1, as expected by their predicted protein sizes (31 kDa for Bmh2 and 30 kDa for Bmh1).

We next asked if Sps1 could bind to Bmh1 and Bmh2, and found that we can co-immunoprecipitate Bmh1 and Bmh2 with SBP-Sps1 (Figure 4C). However, we do not detect an interaction of Bmh1 and Bmh2 with SBP-Sps1-T12A (Figure 4C). These results indicate that Sps1 interacts with Bmh1 and Bmh2 and that T12 within Sps1 is required for the interaction.

### Bmh1 and Bmh2 are expressed during sporulation and are present in the nucleus and the cytoplasm

**Figure 5:**
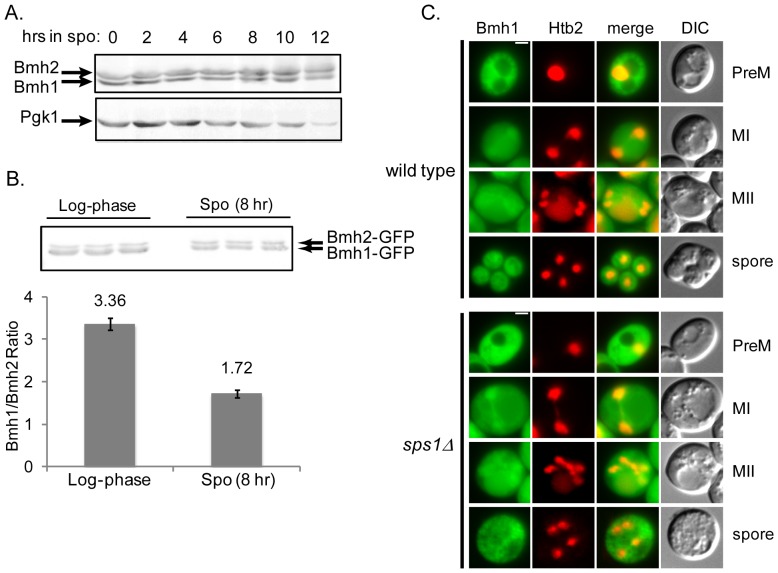
Bmh1 and Bmh2 protein expression and localization during sporulation. (A) Immunoblot probed with anti-Bmh antibody, showing Bmh1 and Bmh2 expression during sporulation using LH177 (*wild type*). Pgk1 was used as a loading control. (B) Quantification of Bmh isoform ratio during log-phase growth and sporulation. LH971 (*BMH1-GFP BMH2-GFP*) was sampled during log-phase growth and at 8 hours into sporulation. Immunoblot was probed with anti-GFP antibody and band intensities were measured. (C) Localization of Bmh1-GFP in a wild type background (LH972; top) and in an *sps1Δ* background (LH974; bottom) during sporulation. Htb2-mCherry is used as a nuclear marker. PreM: Pre-meiosis, MI: Meiosis I, MII: Meiosis II, Spore: mature spore. Scale bar = 2 µ.

We examined Bmh1 and Bmh2 expression during sporulation in wild type cells, and found that both are expressed throughout sporulation (Figure 5A). As Bmh1 was previously reported to be the major isoform in vegetative growth [35], [65], we compared the levels of expression of Bmh1 and Bmh2 during log-phase growth and sporulation. We used the strain *BMH1-GFP BMH2-GFP* and examined the protein levels using an anti-GFP antibody to avoid any bias in isoform detection by the anti-Bmh antibody. We found that the ratio of Bmh1 to Bmh2 in log-phase growth was 3.4±0.2 (mean ± S.D.), decreasing to 1.7±0.2 (mean ± S.D.) in sporulating cells. This suggests that Bmh1 is more abundant than Bmh2 during log-phase growth, but that Bmh2 levels increase relative to Bmh1 in sporulating cells (Figure 5B).

We also examined Bmh1 and Bmh2 localization during sporulation. High-throughput studies have examined Bmh1 and Bmh2 in haploid log-phase cells and found both cytoplasmic and nuclear localization [66]. We examined Bmh1-GFP localization in diploids during both log-phase growth and sporulating cells and saw both nuclear and cytoplasmic localization (Figure 5C, *top*). We see similar results for Bmh2-GFP (Figure S3, *left*). We next asked if 14-3-3 localization is affected in an *sps1Δ* background. We see both nuclear and cytoplasmic localization in the absence of *SPS1* (Figure 5C, *bottom* and Figure S3, *middle*), suggesting that *SPS1* is not required for proper 14-3-3 localization. Finally, we asked if 14-3-3 localization was dependent on the presence of both isoforms. To answer this question, we examined *BMH1-GFP* in a *bmh2Δ* background and we saw no obvious difference in localization in sporulating cells (Figures S3 and S4).

### Yeast deficient in 14-3-3 isoforms exhibit reduced sporulation efficiency

Because we see a defect in sporulation efficiency in cells carrying the *sps1-T12A* allele, we asked if 14-3-3s play a role in sporulating cells. We examined strains that lack either *BMH1* or *BMH2* (LH980: *bmh1Δ/bmh1Δ* and LH981: *bmh2Δ/bmh2Δ*) and saw that both had slight reductions in sporulation efficiency (Table 3). Cells lacking *BMH1* form spores at 71.0%, while cells lacking *BMH2* form spores 70.8% of the time, compared to a wild type value of 80.6% (P<0.02 and P<0.03 respectively compared to wild type, Student's *t* test). To examine whether the slight sporulation efficiency defect in each *bmh* mutant was due to the missing 14-3-3 isoform, or the reduction in the total number of 14-3-3 genes, we examined sporulation in the doubly heterozygous LH979 strain (*bmh1Δ/+ bmh2Δ/+*), which sporulated at 79.6%, similar to wild-type (Table 3).

**Table 3:** *BMH1* and *BMH2* are required for sporulation.

strain name	relevant genotype	% spores (mean ± S.D.)
LH902	wild type	80.7±2.1
LH980	*bmh1Δ/bmh1Δ*	71.0±3.8
LH981	*bmh2Δ/bmh2Δ*	70.8±4.5
LH979	*bmh1Δ/+ bmh2Δ/+*	79.7±4.8
LH982	*bmh1Δ/bmh1Δ bmh2Δ/+*	55.8±8.3
LH983	*bmh1Δ/+ bmh2Δ/bmh2Δ*	50.0±2.5

200 cells/culture and a minimum of 3 cultures were tested for each strain.

We also examined the effects of further reductions in 14-3-3 gene dosage. Although a strain lacking both *bmh1Δ* and *bmh2Δ* was viable in the SK1 strain background, this strain displayed severe growth defects and was unable to enter meiosis (likely because of an inability to successfully grow in YPA pre-sporulation medium, as 14-3-3s have been shown to be required for growth in media containing acetate [33]), precluding our ability to assay the sporulation efficiency of the *bmh1Δ bmh2Δ* double mutant strain. However, we were able to examine sporulation in LH982 (*bmh1Δ/bmh1Δ bmh2Δ/+*) and LH983 (*bmh1Δ/+ bmh2Δ/bmh2Δ*), and found that the spore efficiency defect became more severe as the gene dosage of 14-3-3 was further reduced. Specifically, we see that *bmh1Δ/bmh1Δ bmh2Δ/+* and *bmh1Δ/+ bmh2Δ/bmh2Δ* produced 55.8% and 50.0% spores respectively (both P<0.01 compared to wild type, Student's *t* test). Taken together, these results suggest a role for 14-3-3 proteins in sporulation efficiency (Table 3).

### *SPS1* genetically interacts with the 14-3-3 isoforms, *BMH1* and *BMH2*

Given the physical interaction between Sps1 and the 14-3-3 proteins, and as both display sporulation efficiency defects, we tested whether *SPS1* genetically interacts with *BMH1* and *BMH2*. Consistent with a genetic interaction between the *14-3-3*'s and *SPS1*, LH902 wild type (80.6%), LH979 *bmh1Δ/+ bmh2Δ/+* (79.6%), and LH965 *sps1Δ/+* (81.0%) all show similar levels of sporulation, while LH984 (*bmh1Δ/+ bmh2Δ/+ sps1Δ/+*) showed a reduction in spore formation, forming spores 67.3% of the time (P<0.03 compared to wild type, Student's *t* test) (Tables 3 and 4).

**Table 4:** *SPS1* genetically interacts with *BMH1* and *BMH2*.

strain name	relevant genotype	% spores (mean ± S.D.)
LH902	wild type	80.7±2.1
LH984	*bmh1Δ/+ bmh2Δ/+ sps1Δ/+*	67.3±7.8
LH965	*sps1Δ/+*	81.0±5.4
LH985	*bmh1Δ/+ bmh2Δ/+ SBP-sps1-T12A/sps1Δ*	22.5±3.9
LH970	*SBP-sps1-T12A/sps1Δ*	53.0±6.5

200 cells/culture and a minimum of 3 cultures were tested for each strain.

If mutation of threonine 12 to alanine was sufficient to completely abrogate the interaction of the 14-3-3 proteins and Sps1, then LH985 (*bmh1Δ/+ bmh2Δ/+ SBP-sps1-T12A/sps1Δ*) should have a spore efficiency defect equivalent to LH970 (*SBP-sps1-T12A/sps1Δ*), since the lack of the T12 should eliminate the Sps1 14-3-3 interaction. Instead, we found that LH985 (*bmh1Δ/+ bmh2Δ/+ SBP-sps1-T12A/sps1Δ*) sporulates at an efficiency of 22.5%, which is less efficient than LH970 (*SBP-sps1-T12A/sps1Δ*), which forms spores at an efficiency of 53.0% (P<0.01 compared to *SBP-sps1-T12A/sps1Δ*, Student's *t* test). This result suggests that T12 of Sps1 is not the sole mediator of its interaction with Bmh1 and Bmh2 (Table 4).

### Localization of Sps1 and Sps1-T12A during sporulation

Having shown that Sps1, Bmh1 and Bmh2 are all expressed at the same time during sporulation and that they are capable of physically interacting, we investigated if Sps1 localization resembled that of the 14-3-3s. While a previous report suggested an N-terminal GFP-Sps1 fusion contained within strain Y5050 [11] localizes to the prospore membrane, we were unable to detect such localization. Amplification and sequencing of the *GFP-SPS1* locus from Y5050 revealed that the start codon (ATG) was absent from the construct, indicating that it does not produce the intended protein product (Figure S5).

**Figure 6:**
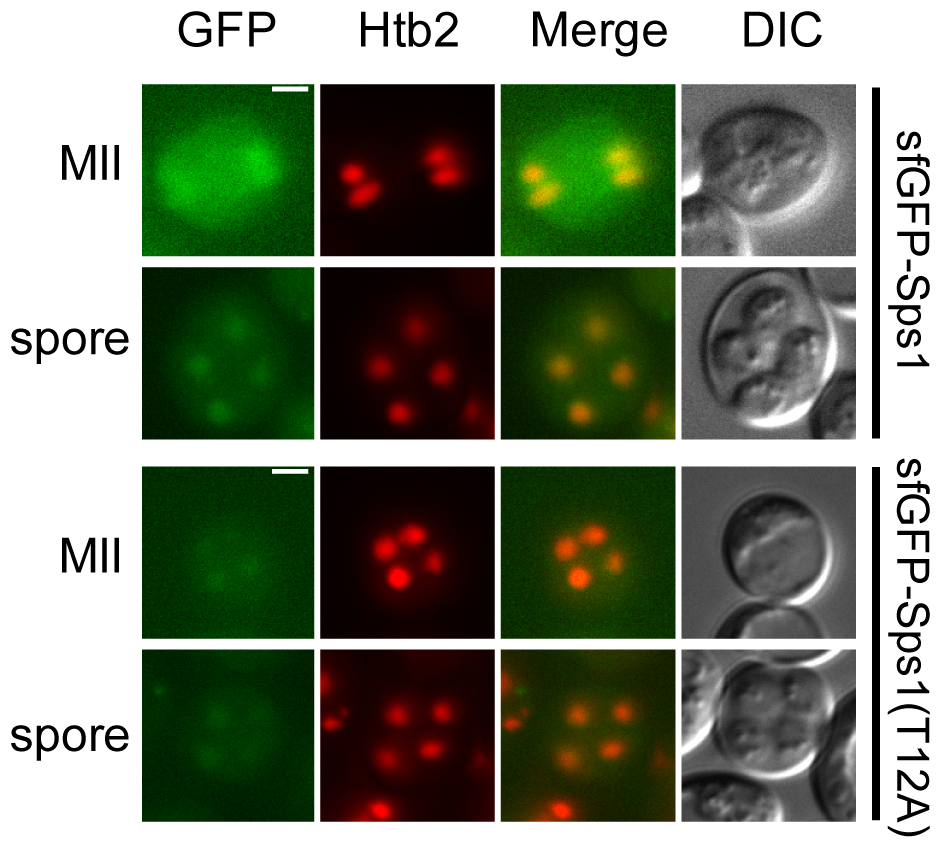
Localization of Sps1 and Sps1-T12A during sporulation. Localization of sfGFP-Sps1 (LH986; top) and sfGFP-Sps1-T12A (LH987; bottom) during sporulation. Htb2-mCherry is used as a nuclear marker. MII: Meiosis II, Spore: mature spore. Scale bar = 2 µ.

To determine Sps1 localization, we constructed an N-terminal *sfGFP-SPS1* fusion by integrating a monomeric variant of the fast-folding *superfolderGFP* (*sfGFP*) [67]–[69] at the *SPS1* locus. We see sfGFP-Sps1 in both the cytoplasm and the nucleus (Fig. 6 *top*). As spores matured and became refractile, nuclear localization became more distinct and cytoplasmic localization was reduced, compared to cells earlier in the sporulation process (Fig. 6 *top*). As 14-3-3 proteins are known to affect the localization of their target proteins, we wanted to determine if mutation of threonine 12 to alanine affected Sps1 localization. Using *sfGFP-sps1-T12A*, we saw a similar pattern of localization as we did in *sfGFP-SPS1* (Figure 6 *bottom*), suggesting that 14-3-3 proteins do not affect Sps1 localization.

### Sps1 has a nuclear localization sequence

**Figure 7:**
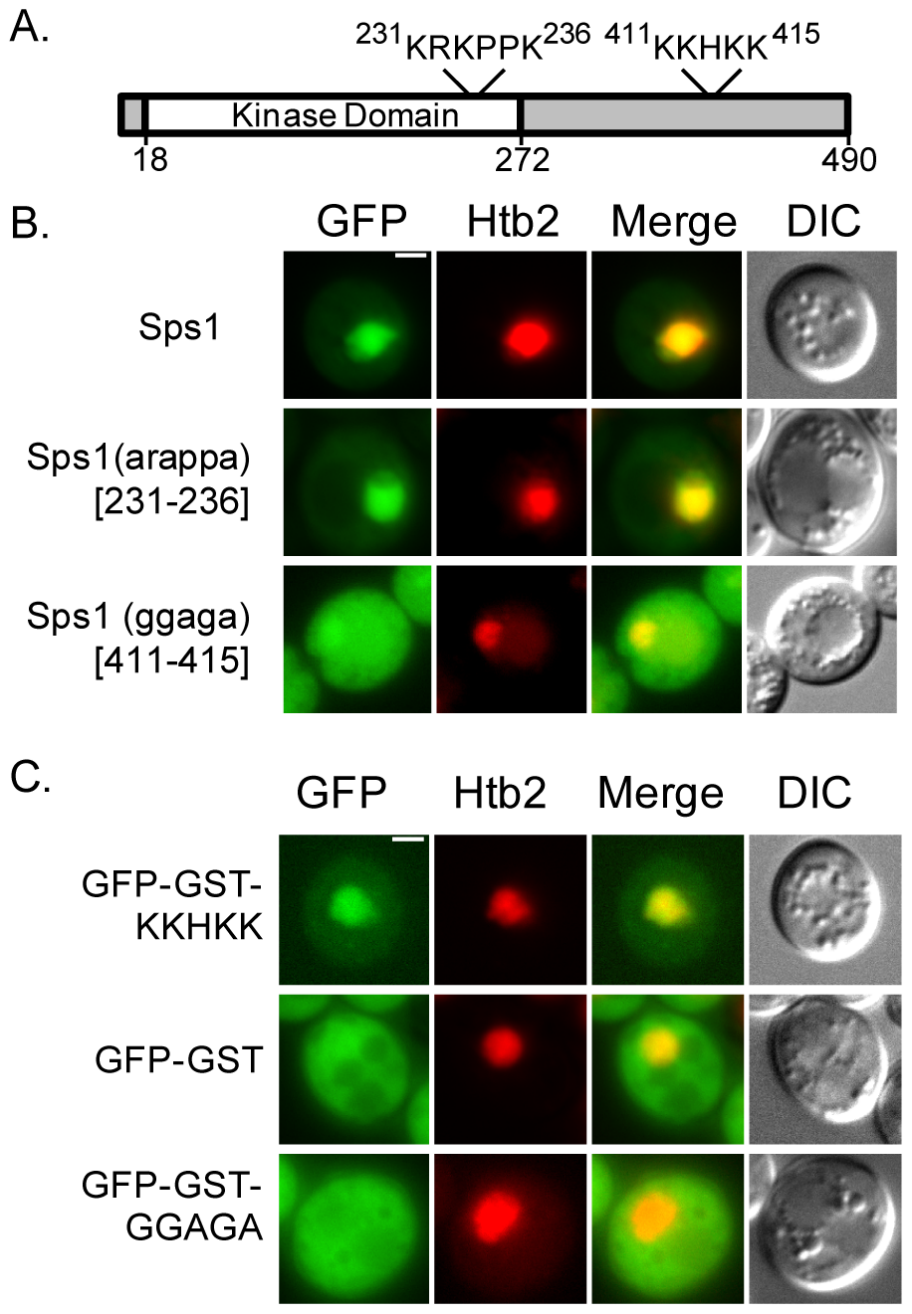
Sps1 has a nuclear localization sequence. (A) Diagram of Sps1 indicating potential nuclear localization signals. The first KRKPPK (231–236) lies within the kinase domain and the second, KKHKK (411–415) is located toward the C-terminus. (B) The amino acid sequence, KKHKK (411–415) is necessary for proper nuclear localization of Sps1. LH995 (*pGFP-SPS1*), *top*, LH997 (*pGFP-sps1-arappa (213–236)*), *middle*, and LH996 (*pGFP-sps1-ggaga (411–415)*), *bottom*, were visualized during log-phase growth; Htb2-mCherry is used as a nuclear marker. (C) The NLS of Sps1 (KKHKK (411–415)) is sufficient to localize a GFP-GST construct to the nucleus. LH999 (p*GFP-GST-SPS1(387–438)*), *top*, LH998 (p*GFP-GST*), *middle*, and LH1000 (p*GFP-GST-sps1-ggaga-(387–438)*), *bottom*, were visualized during log-phase growth; Htb2-mCherry is used as a nuclear marker. Scale bar = 2 µ.

As we observed sfGFP-Sps1in the nucleus, we examined the amino acid sequence of Sps1 and found two putative nuclear localization sequences (NLS) based on their similarity to the classical SV40 monopartite NLS [70] (Figure 7A). We compared the localization of GFP-Sps1 with GFP-tagged alleles in which each putative NLS was mutated (pRS426-*GFP-SPS1*, pRS426-*GFP-sps1-arappa* and pRS426-*GFP-sps1-ggaga*). We visualized these different fusion proteins in log-phase cells expressing Sps1 and mutant variants ectopically, using the *TEF2* promoter on a multicopy vector (Fig. 7B). We found that changing residues 231–236 KRKPPK to ARAPPA had no effect on nuclear localization (Fig. 7B, *middle row*). However, mutation of residues 411–415 KKHKK to GGAGA prevented distinct nuclear localization (Fig. 7B, *bottom row*). This experiment demonstrated the necessity of residues 411–415 (KKHKK) for proper nuclear localization.

We then asked whether this sequence was sufficient to drive the nuclear localization of a protein that is normally not present in the nucleus. A GFP-GST fusion was created, in which the addition of GST prevents the passive diffusion into the nucleus of GFP [71]. We observed an increase in nuclear localization of the fusion protein containing an intact NLS in comparison to those with just the GFP-GST fusion or a GFP-GST fusion with mutated NLS (Fig. 7C). Therefore, Sps1 has a functioning NLS that is both necessary and sufficient for nuclear localization.

## Discussion

Sps1 is a serine/threonine kinase that is expressed during sporulation and functions in the process of spore formation. Interestingly, genome-wide studies have identified targets of yeast 14-3-3 proteins [16], [72], although none of these studies have identified Sps1 as a target for either Bmh1 or Bmh2.

In this study, we use phylogeny to show that Sps1 is a bona-fide GCKIII kinase, and identify three regions important for its function: a conserved C-terminal motif containing the invariant ExxxPG at residues 464–469, a phosphorylation site T12, and a nuclear localization signal from 411 to 415 (KKHKK). We also show that T12 is part of a 14-3-3 binding site, and is required for the physical interaction between Sps1 and the 14-3-3 isoforms Bmh1 and Bmh2. We demonstrate that 14-3-3s are important for sporulation, as cells lacking in *BMH1* and *BMH2* have defects in sporulation efficiency and show a genetic interaction with *SPS1*. Because we see both a genetic and physical interaction, we propose that *SPS1* and the 14-3-3s act together to regulate sporulation.

### Sps1 is a GCKIII family member

While an initial analysis suggested Sps1 was an outlier with respect to the Ste20 family [5], our bioinformatic analyses indicate that Sps1 belongs to the GCKIII family of Ste20 kinases. The identification of Sps1 as a GCKIII emerged using two different phylogenetic reconstruction strategies, supporting the robustness of the determination. The phylogenetic results also indicate that Sps1 is present in a distinct clade of animal and plant GCKIII kinases, indicating that the GCKIII kinases are an ancient and distinct subfamily of Ste20 kinases, likely present in the common eukaryotic ancestor ∼1 billion years ago.

Our comparison of Sps1 with other GCKIII kinases led to the identification of a previously unappreciated conserved region found in all GCKIII kinases. This region is found at the C-terminus of the regulatory domain, and we call this region the EPG motif. Our experimental results demonstrate the importance of this region and suggest that the EPG motif may play an important role in other GCKIII kinases.

We also identify a nuclear localization sequence on Sps1, which is both necessary and sufficient for directing nuclear localization (residues 411–415). Other GCKIII proteins have also been reported to localize to both the nucleus and cytoplasm, and the nuclear localization domain of Mst3 has been mapped to residues 278–294 [73]; the location of this nuclear localization signal is conserved in the mammalian GCKIIIs and in *Drosophila*, but has changed in Sps1.

### Role of *BMH1* and *BMH2* in sporulation

Here we show that *BMH1* and *BMH2* are important for the efficient formation of spores in the SK1 background. Previous studies have not identified a role in sporulation for *BMH1*, including a genome-wide study using the yeast deletion collection in the S288c background [74]. It is possible that loss of *BMH1* and *BMH2* does not have a sporulation defect in the S288c background, or it could be that the less efficiently sporulating S288c strain background made it more difficult to detect the mild sporulation defect we see in *bmh1* and *bmh2* mutants.

In most yeast strain backgrounds examined, the *bmh1 bmh2* double mutant is inviable. In the SK1 strain, the *bmh1 bmh2* mutant is viable, but grows very slowly and produces cells of abnormal morphology, precluding the ability to examine the double mutant during sporulation. Because of this, we examined sporulation in a *bmh1/+ bmh2/bmh2* strain and see that this strain sporulates at 50% (compared to wild type levels of about 81%), a more severe phenotype than that seen with either single mutant. Our results suggest that if we were able to examine the sporulation defect in a *bmh1 bmh2* double mutant, we may see a more severe sporulation efficiency of less than 50%.

Why are there two 14-3-3 isoforms in yeast? In higher eukaryotes, there are several isoforms of 14-3-3 proteins, and these different isoforms are hypothesized to play different roles [28], [75]. The yeast Bmh1 and Bmh2 are considered paralogs, and likely arose during a whole genome duplication event [76]. The yeast 14-3-3 isoforms appear to be largely redundant in terms of function, although specific phenotypes have been associated with the loss of only a single isoform. For instance, the loss of *BMH1* causes an increase in glycogen accumulation [77] whereas loss of *BMH2* results in abnormal accumulation of polyphosphate [78].

We see that 14-3-3 isoform levels shift during sporulation. In vegetatively growing cells, we see that Bmh1 is more prevalent compared to Bmh2, as previously described [65]. However, during sporulation, Bmh2 levels rise in comparison to Bmh1 levels. We do not know whether this relative increase in Bmh2 level is important for 14-3-3 function or is merely a consequence of transcriptional regulation changes that occur during sporulation.

### 14-3-3 regulation of Sps1 during sporulation

Our data suggests that Bmh1 and Bmh2 are important for the positive regulation of Sps1 function during sporulation, since the phenotypes of the strains containing *sps1-T12A* are similar to those lacking *bmh1*, and *bmh2*. As Bmh1 and Bmh2 physically interact with Sps1 in a manner that depends on the T12 within Sps1, these data raise the possibility that this interaction is functionally significant.

We propose that the interaction with 14-3-3s modulates Sps1 function but is not absolutely required for Sps1 activity, because an *sps1* null allele has a much more severe phenotype than either the *sps1-T12A* or the *bmh1* or *bmh2* mutants. In comparison to cells carrying *sps1Δ* allele, which rarely produce refractile spores, cells with the *sps1-T12A* allele display a less severe phenotype, sometimes producing spores that appear normal with respect to their ability to form the outer layers of the spore wall, to package the appropriate number of spores within an ascus, and to germinate.

Although we cannot directly assay the phenotype of the *bmh1 bmh2* double mutant, we anticipate that it would be more severe than the *sps1-T12A* phenotype because cells carrying *sps1-T12A* allele sporulate more efficiently than the *bmh1/+ bmh2/bmh2* mutant cells (Tables 2 and 3). The more severe defect seen in the *bmh1/+ bmh2/bmh2* mutant along with more severe defect seen in *bmh1Δ/+ bmh2Δ/+ SBP-sps1-T12A/sps1Δ* compared to *SBP-sps1-T12A/sps1Δ* (Tables 3 and 4) suggests that Sps1 may not be the only relevant binding partner of 14-3-3 proteins during sporulation. Alternatively, it is possible that T12 is not the only residue on Sps1 that can mediate the Sps1-14-3-3 interaction. It is possible that the weak phenotype seen with *sps1-T12A* compared to the *bmh1/+ bmh2/bmh2* mutant is due to the presence on Sps1 of other 14-3-3 interaction sites.

### 14-3-3 regulation of other GCKIII family kinases

The modulation of GCKIII kinases by 14-3-3s may be evolutionarily conserved. First, a high-throughput screen identified a physical interaction between the mammalian GCKIII Mst4 and 14-3-3ε [79]. Second, both MST4 and YSK1/SOK1 have predicted 14-3-3 binding motifs C-terminal to their kinase regions (Scansite). Third, the brain specific isoform of MST3 has been shown to be phosphorylated on threonine 18 [80], which is within a 14-3-3 consensus binding site, though binding with 14-3-3s has yet to be demonstrated.

Other interactions with 14-3-3 proteins and GCKIII kinases have been identified. 14-3-3ζ is a substrate of YSK1/SOK1, and its phosphorylation has been shown to be important for Golgi positioning [81] and to disrupt the binding of the proapoptotic factor ASK to14-3-3ζ during apoptosis [82].

Interestingly, there may be a role for 14-3-3s in modulating Ste20 family kinases in general. In the Σ1278 background, 14-3-3s have been found to bind directly to the C-terminal kinase-containing portion (from amino acid 494 to 939) of the Ste20 kinase, a PAK family member [37]. Since the domain architecture of PAKs and GCKs are reversed, and since Bmh1 and Bmh2 interacts with the C-terminal half of Ste20 and the N-terminal half of Sps1 (which are both STE20 family kinases), it is tempting to speculate that Bmh1 and Bmh2 may modulate pseudohyphal-development directly through the modulation of Ste20 activity, especially because our analysis of amino acids 494–939 of Ste20 using Scansite predicts a 14-3-3 binding site of medium stringency surrounding threonine 546, which has been found to be phosphorylated by a number of different studies [83]–[85].

## Supporting Information

Figure S1 **Analysis of Sps1 protein.** (A) Lysates from LH791 (*SPS1-zz*) and LH953 (*sps1ΔEPG-zz*) were collected throughout sporulation. Immunoblots were probed with rabbit antisera. (B) Lysates from LH954 (*SBP-SPS1*) and LH955 (*SBP-sps1-T12A*) were collected throughout sporulation. Immunoblots were probed with anti-SBP antibody. Pgk1 was used as a loading control in both (A) and (B).
(TIF)

Figure S2 **Analysis of** ***sps1-T12A*** **spores.** (A) Quantification of the number of spores formed per ascus for (left to right): LH902 (*WT*), LH962 (*SBP-SPS1*) and LH968 (*SBP-sps1-T12A*) and LH970 (*SBP-sps1-T12A/sps1Δ*). (B) Dityrosine assay examining the outer spore wall layer. Visible light image of nitrocellulose membrane with yeast cell patches, *left*; UV light image of the same membrane, *right*. Strains shown are LH956 (*dit1Δ*), LH177 (*WT*), LH872 (*sps1Δ*), LH955 (*SBP-sps1-T12A*) and LH954 (*SBP-SPS1*). (C) Spore wall permeability assay. Impermeable spore with ssGFP correctly localized to the spore wall, *left*, permeable spore that has incorrectly allowed ssGFP to disperse between the spore wall and ascal membrane, *right*. LH954 (*SBP-SPS1*) and LH955 (*SBP-sps1-T12A*) were transformed with pRS424-ssGFP. The permeability of the spore was scored for each strain.
(TIF)

Figure S3 **Bmh2 localization during sporulation does not depend on** ***SPS1*** **or** ***BMH1*** **.** Localization during sporulation of Bmh2, as seen in LH973 (*BMH2-GFP*), *left*, LH975 (*BMH2-GFP sps1Δ*), *middle*, and LH978 (*BMH2-GFP bmh1Δ*), *right*. Htb2-mCherry is used as a nuclear marker. PreM: Pre-meiosis, MI: Meiosis I, MII: Meiosis II, Spore: mature spore. Scale bar = 2 µ.
(TIF)

Figure S4 **Bmh1 localization during sporulation does not depend on** ***BMH2*** **.** Localization during sporulation of Bmh1, as seen in LH977 (*BMH1-GFP bmh2Δ*). Htb2-mCherry is used as a nuclear marker. PreM: Pre-meiosis, MI: Meiosis I, MII: Meiosis II, Spore: mature spore. Scale bar = 2 µ.
(TIF)

Figure S5 **Sequencing of Y5050 reveals no start codon.** Sequencing results of the Sps1 locus from Y5050 [11] reveals the lack of a start codon before GFP.
(TIF)

Table S1 **Plasmids used in this study.**
(PDF)

Table S2 **Primers used in this study.**
(PDF)

Table S3 ***S. cerevisiae*** **strains used in this study.**
(PDF)
