# Supplementary figures and images for: The GCKIII Kinase Sps1 and the 14-3-3 Isoforms, Bmh1 and Bmh2, Cooperate to Ensure Proper Sporulation in Saccharomyces cerevisiae
Source: PLoS One. 2014 Nov 19;9(11):e113528. doi: 10.1371/journal.pone.0113528 (PMC4237420; doi:10.1371/journal.pone.0113528)

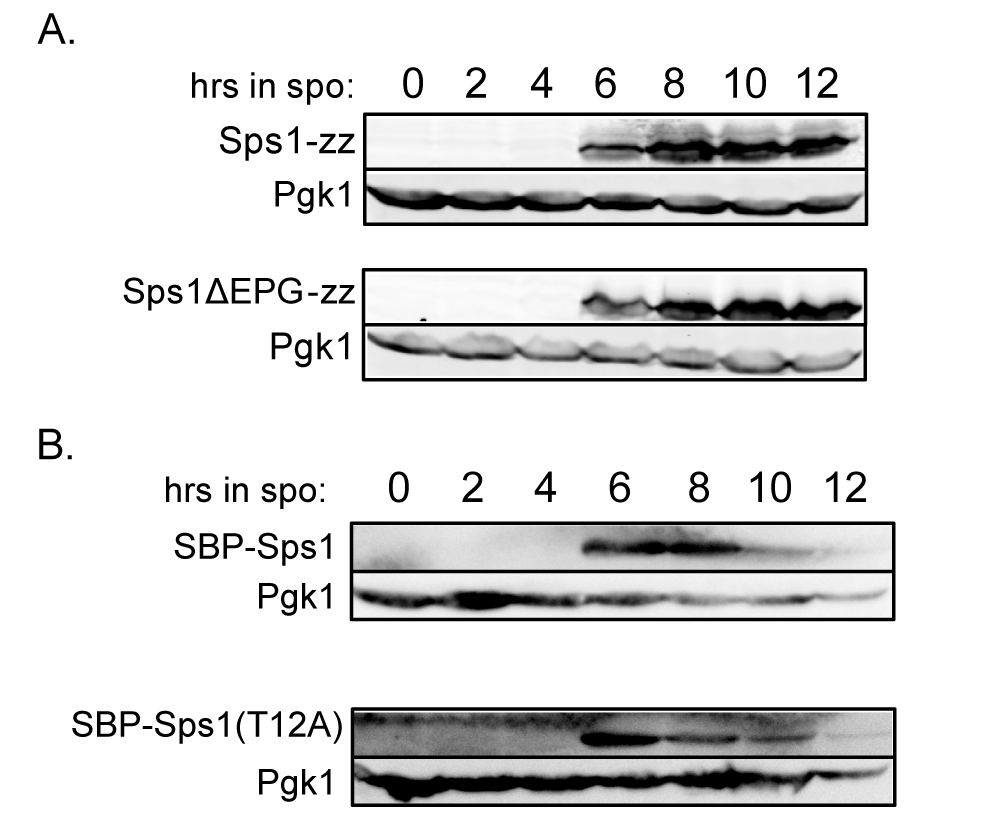

Supplement: Figure S1 — Analysis of Sps1 protein. (A) Lysates from LH791 (SPS1-zz) and LH953 (sps1ΔEPG-zz) were collected throughout sporulation. Immunoblots were probed with rabbit antisera. (B) Lysates from LH954 (SBP-SPS1) and LH955 (SBP-sps1-T12A) were collected throughout sporulation. Immunoblots were probed with anti-SBP antibody. Pgk1 was used as a loading control in both (A) and (B). (TIF) [file pone.0113528.s001.tif]

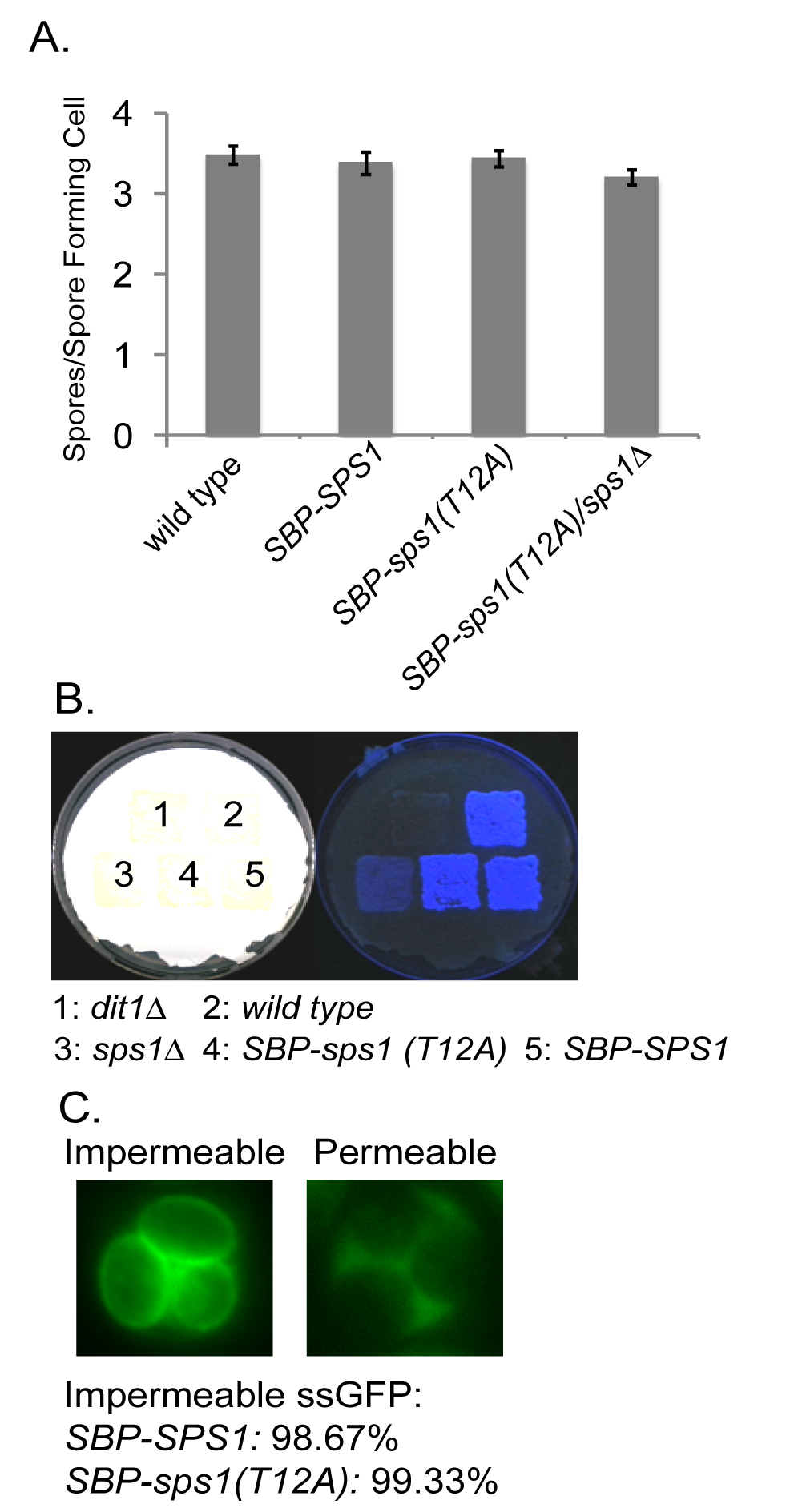

Supplement: Figure S2 — Analysis of sps1-T12A spores. (A) Quantification of the number of spores formed per ascus for (left to right): LH902 (WT), LH962 (SBP-SPS1) and LH968 (SBP-sps1-T12A) and LH970 (SBP-sps1-T12A/sps1Δ). (B) Dityrosine assay examining the outer spore wall layer. Visible light image of nitrocellulose membrane with yeast cell patches, left; UV light image of the same membrane, right. Strains shown are LH956 (dit1Δ), LH177 (WT), LH872 (sps1Δ), LH955 (SBP-sps1-T12A) and LH954 (SBP-SPS1). (C) Spore wall permeability assay. Impermeable spore with ssGFP correctly localized to the spore wall, left, permeable spore that has incorrectly allowed ssGFP to disperse between the spore wall and ascal membrane, right. LH954 (SBP-SPS1) and LH955 (SBP-sps1-T12A) were transformed with pRS424-ssGFP. The permeability of the spore was scored for each strain. (TIF) [file pone.0113528.s002.tif]

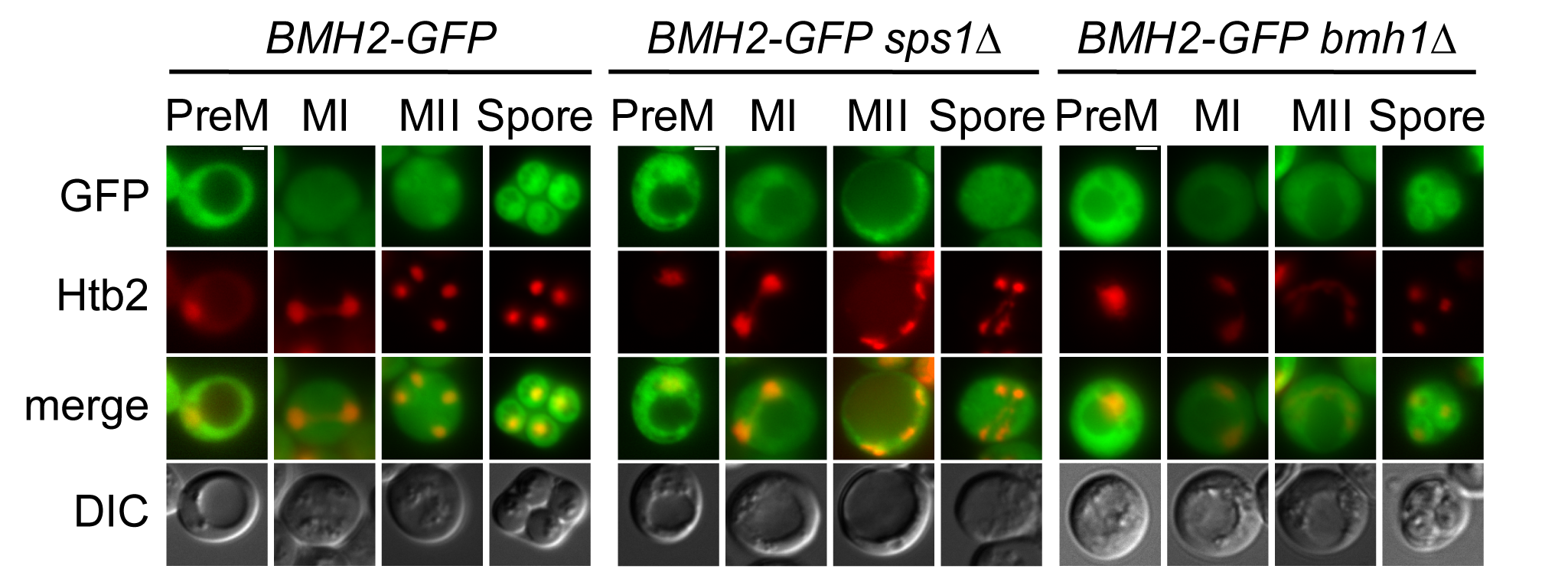

Supplement: Figure S3 — Bmh2 localization during sporulation does not depend on SPS1 or BMH1 . Localization during sporulation of Bmh2, as seen in LH973 (BMH2-GFP), left, LH975 (BMH2-GFP sps1Δ), middle, and LH978 (BMH2-GFP bmh1Δ), right. Htb2-mCherry is used as a nuclear marker. PreM: Pre-meiosis, MI: Meiosis I, MII: Meiosis II, Spore: mature spore. Scale bar = 2 µ. (TIF) [file pone.0113528.s003.tif]

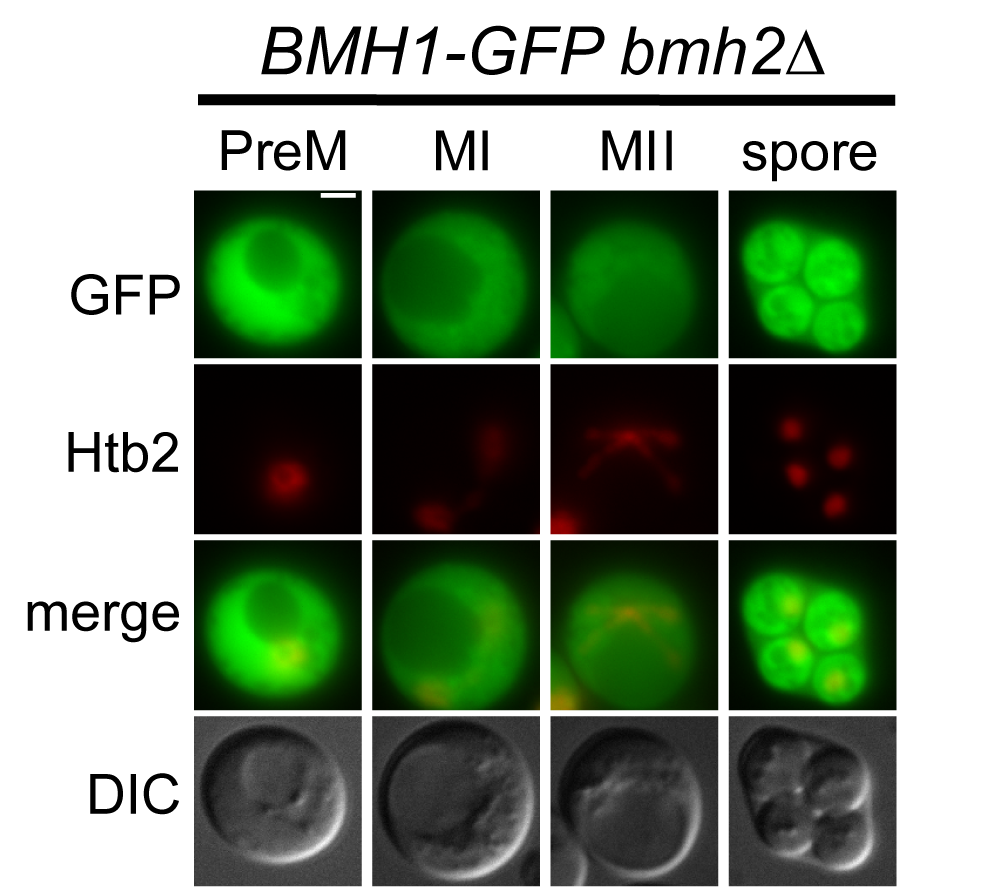

Supplement: Figure S4 — Bmh1 localization during sporulation does not depend on BMH2 . Localization during sporulation of Bmh1, as seen in LH977 (BMH1-GFP bmh2Δ). Htb2-mCherry is used as a nuclear marker. PreM: Pre-meiosis, MI: Meiosis I, MII: Meiosis II, Spore: mature spore. Scale bar = 2 µ. (TIF) [file pone.0113528.s004.tif]

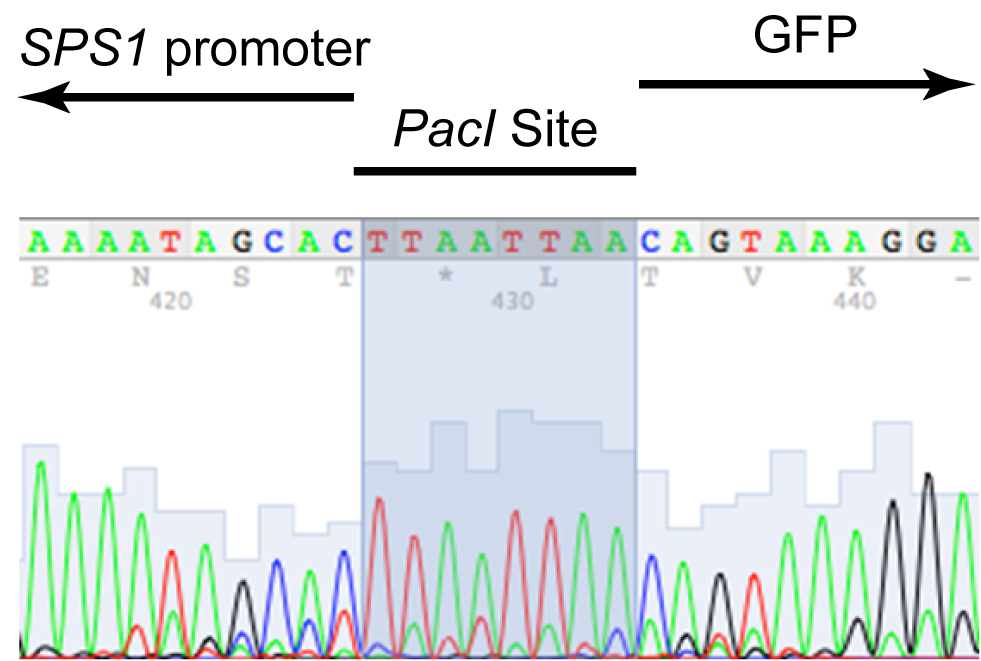

Supplement: Figure S5 — Sequencing of Y5050 reveals no start codon. Sequencing results of the Sps1 locus from Y5050 [11] reveals the lack of a start codon before GFP. (TIF) [file pone.0113528.s005.tif]
