# Supplementary material for: The GCKIII Kinase Sps1 and the 14-3-3 Isoforms, Bmh1 and Bmh2, Cooperate to Ensure Proper Sporulation in Saccharomyces cerevisiae
Source: PLoS One. 2014 Nov 19;9(11):e113528. doi: 10.1371/journal.pone.0113528 (PMC4237420; doi:10.1371/journal.pone.0113528)
Supplement: Table S1 — Plasmids used in this study. (PDF) [file pone.0113528.s006.pdf]

SUPPORTING TABLE S1: Plasmids used in this study

Plasmid	Description	Source
pCgW	YIp *TRP1*$^{C.g.}$	Kenji Irie
pHIS3	YIp *HIS3*$^{C.g.}$	Parodi *et. al.* 2012
pLEU2	YIp *LEU2*$^{C.g.}$	Kenji Irie
pURA3	YIp *URA3*$^{K.l.}$	Kenji Irie
pFA6a-GFP(S65T)-TRP1	YIp *GFP-TRP1*	Longtine *et.al*., 1998
pFA6a-13Myc-TRP1	YIp *13xMyc-TRP1*	Longtine *et.al*., 1998
pBS1365	YIp *zz-URA3*$^{K.l.}$	Puig *et.al*., 1998
pDHL1029	pUC19-linker-msfGFP-FRT Kan FRT-Amp	Dirk Landgraf
pRS424-ssGFP	YEp P_{SPR1}-*SPR1signalsequence-GFP-TRP1*	Suda *et. al.* 2009
pMK33-CTAP(SG)	Expression vector for Drosophila cell culture	Kyriakakis *et. al.*
pGEX-4T-3	GST containing bacterial expression plasmid	GE Healthcare Life Sciences
pRS316	YCp, URA3 selectable	Sikorski and Hieter 1989
pRS426-G71(1-1245)	YEp P_{TEF2}-*GFP-SPO71(1-1245)-URA3*	Parodi *et. al.* 2012

pCS22	YEp P_{TEF2}-*GFP-SPS1-URA3*	This Study
pCS65	YEp P_{TEF2}-*GFP-sps1-ggaga-URA3*	This Study
pCS130	YCp P_{TEF2}-*GFP-sps1-arappa-URA3*	This Study
pCS75	YEp P_{TEF2}-*GFP-GST-URA3*	This Study
pCS60	YEp P_{TEF2}-*GFP-GST-SPS1(387-438)-URA3*	This Study
pCS78	YEp P_{TEF2}-*GFP-GST-sps1-ggaga-(387-438)URA3*	This Study
pCS20	YEp P_{TEF2}-*SBP-SPS1-URA3*	This Study
pCS28	YEp P_{TEF2}-*SBP-sps1-K47R-URA3*	This Study
pCS96	YCp P_{TEF2}-*SBP-SPS1-URA3*	This Study
pCS98	YCp P_{TEF2}-*SBP-sps1-T12A-URA3*	This Study
pCS107	YCp P_{TEF2}-*SBP-URA3*	This Study
pCS99	YEp P_{SPS1}-*SBP-SPS1-URA3*	This Study
pCS47	pBSIIKS+ with *SPS1* coding sequence, including 137 bps upstream and 297 bps downstream, cloned between ClaI and SpeI restriction sites	This Study
pCS159	pCS47 with *SPS1* mutated to *sps1-S345A*	This Study

pCS100	YCp P_{SPS1}-*SBP-sps1-T12A-URA3*	This Study
pCS146	YCp P_{SPS1}-*sfGFP-SPS1-URA3*	This Study
pCS145	YCp P_{SPS1}-*sfGFP-sps1-T12A-URA3*	This Study

YIp: Yeast Integrating Plasmid

YEp: Yeast Episomal Plasmid

YCp: Yeast Centromere Plasmid

References:

Kyriakakis P, Tipping M, Abed L, Veraksa A 2008. Tandem affinity purification in Drosophila: the advantages of the GD-TAP system. Fly 2(4):229-235.

Longtine MS, McKenzie III A, Demarini DJ, Shah NG, Wach A, Brachat A, Philippsen P, Pringle JR 1998. Additional modules for versatile and economical PCR-based gene deletion and modification in Saccharomyces cerevisiae. Yeast 14:953–961.

Parodi EM, Baker CS, Tetzlaff C, Villahermosa S, Huang LS 2012. SPO71 Mediates Prospore Membrane Size and Maturation in Saccharomyces cerevisiae. Eukaryot. Cell 11(10):1191-1200.

Puig O, Rutz B, Luukkonen BG, Kandels-Lewis S, Bragado-Nilsson E, Séraphin B 1998. New constructs and strategies for efficient PCR-based gene manipulations in yeast. Yeast 14:1139–1146.

Sikorski RS, Hieter P 1989. A system of shuttle vectors and yeast host strains designed for efficient manipulation of DNA in Saccharomyces cerevisiae. Genetics 122(1):19-27.

Suda Y, Rodriguez RK, Coluccio AE, Neiman AM 2009. A screen for spore wall permeability mutants identifies a secreted protease required for proper spore wall assembly. PLoS One 4(9):e7184.
