# Supplementary material for: The GCKIII Kinase Sps1 and the 14-3-3 Isoforms, Bmh1 and Bmh2, Cooperate to Ensure Proper Sporulation in Saccharomyces cerevisiae
Source: PLoS One. 2014 Nov 19;9(11):e113528. doi: 10.1371/journal.pone.0113528 (PMC4237420; doi:10.1371/journal.pone.0113528)
Supplement: Table S2 — Primers used in this study. (PDF) [file pone.0113528.s007.pdf]

SUPPORTING TABLE S2: Primers used in this study

**Primer ID#**	**Sequence** (All sequences listed as 5'→3')
OLH95	GTTTAAACGAGCTCGAATTC
OLH99	GGTCATAGCTGTTTCCTGTG
OLH131	CTAGCTAATTCATTTACTAAACAACAAAAGAAAATAGC ACAAACACAGGAAACAGCTATGACC
OLH132	ACTCATGCATATACACACATTATATATATATATCTATTT TTTTAGTTGTAGTTGTAAAACGACGGCCAGT
OLH389	GAGATCTCACTAAGAATTGAAGCAATAAAGAAAGGATT CGTTGGTCGACGGATCCCCGGGTT
OLH390	CAAAAACTCATGCATATACACACATTATATATATATATC TATTTTTTTATCGATGAATTCGAGCTCGTT
OLH391	GAGATCTCACTAAGAATTGAAGCAATAAAGAAAGGATT CGTTAAGCTGGAGCTCAAAAC
OLH392	CAAAAACTCATGCATATACACACATTATATATATATATC TATTTTTTTATACGACTCACTATAG
OLH487	AGAATGTATGATCGTGCGCGCGACGATGAAACAAGAAA ATACAAGCTGGAGCTCAAAAC
OLH488	AACTCATGCATATACACACATTATATATATATATCTATT TTTTTATACGACTCACTATAGGG
OLH489	CCCTATAGTGAGTCGTATAAAAAAATAGA
OLH490	ATGTTAAAGCAATTCATTAAAACTGAGGC
OLH491	TGCAGCGGATGATTTAATTAACTGC
OLH503	ATATAATTCAATATATCCATCCTTCAT
OLH506	TGTAGGAACGCCGTATTG
OLH507	CACCATAACGAACTTGAA
OLH510	TTATGATTAGAGAGAGGA
OLH512	GTAAGCCCTTGAGTAACT
OLH513	CAAACCGTTGACGTAACT
OLH608	TATCCTAATTCGGTAAAGCTTTGTCGAGACATTAACAAA ACACAGGAAACAGCTATGACC
OLH609	GAACAAAAAGGTAGACCAATGTAGCGCTCTTACTTTAT TAGTTGTAAAACGACGGCCAGT
OLH614	GCGCCTTAAGGGACCTTCTA
OLH615	TGTCGCTTCTGCAATACAGG
OLH710	CGCAAGAAATTGTGGCAATTAGAGTGGTCAACC
OLH711	AATTGCCACAATTTCTTGCGTAACTCTATC
OLH778	CCTGAAAGAAGTGCCTCAGC
OLH780	TTGACGCGAAAGAATGACAC
OLH796	CGAATCCTTTCTTTATTGCTTC
OLH826	TACTAGCTAATTCATTTACTAAACAACAAAAGAAAATA GCACAAACACAGGAAACAGCTATGACC
OLH827	CGATGGAGGGGTTCTTGACCTAATTGATATTTCTTTGCT TTCCATGTTGTAAAACGACGGCCAGT
OLH830	CCTCAAGTTATATTTCGGAGCTG
OLH831	TGACCACCTTAATTGCCACA
OLH1056	GCAGCAAACAACTTCCGGAGCCATCCAATACGGCGTTC CTACAAAGTTGTAAAAC GACGGCCAGT

OLH1128	CAACAAGCTTATGGAAAGCAAAGAAATATCAATTAG
OLH1129	TACACTCGAGTTAAACGAATCCTTTCTTTATTG
OLH1132	AGTAGAATTCATGGACGAGAAGACCACC
OLH1133	CAACAAGCTTAGGCTCGCGTTGCCCCT
OLH1182	TTCCTCTCTCTAATCATAATGGTGGTGCTGGTGCTAACG ATATTC
OLH1183	ATTATGATTAGAGAGAGGAACTATTTCGGTC
OLH1195	TCTTAAGATTGTGTTCAACTGGTTC
OLH1226	TACACTCGAGTTAAGGCTCGCGTTGCCCCT
OLH1230	TGCATTCAAATGTAGATTCAGC
OLH1257	ATTCAGAATTCTTTGTGCTATTTTCTTTTGTTGTTTAG
OLH1258	CTTAAAGCTTATGTCCCCTATACTAGGTTATTG
OLH1259	TTATCTCGAGTTAGACCCGGGAATTCGGGGATC
OLH1260	TTATCTCGAGGACCCGGGAATTCGGGGATC
OLH1261	GATCCCCGAATTCCCGGGTCGGAATGGATATAGATTCA GGCTG
OLH1262	TTATCTCGAGGTTGAGAATATGAGATACAATG
OLH1276	CAACAACAAGCTCCAGCTGAACAAACTCAAGGTGAACC AACCAAACGGATCCCCGGGTTAATTAA
OLH1277	ACTGGAGTGGTAAATCTTCATTTCCCCTTGTATTTCTCA GCGCTCGAATTCGAGCTCGTTTAAAC
OLH1278	CAATTCGTTTAGGTCTAGCTTTG
OLH1280	TCCCTTGTGGTTATTTGACTTTG
OLH1281	AAATATCAATTAGGTCAAGAGCCCCTCCATCGAAGCTA TATTC
OLH1282	TCTTGACCTAATTGATATTTCTTTGCTTTC
OLH1305	CAGCAACAGCAGCCACCTGCTGCCGCCGAAGGTGAAGC ACCAAAG CGGATCCCCGGGTTAATTAA
OLH1307	TTCTGAAATTGCCACCACAG
OLH1308	CGTTTGAATGAAGCAGCAAG
OLH1309	GCAAGTGAGAAGAAAAAGCAAGTTAAAGATAAACTAA AGATAAAACACAGGAAACAGCTATGACC
OLH1311	GAAAAATTATCAAATCAACAAAAAGTACCCGTTACAAC AAAAAAACACAGGAAACAGCTATGACC
OLH1312	ACTGGAGTGGTAAATCTTCATTTCCCCTTGTATTTCTCA GCGCTCGTTGTAAAACGACGGCCAGT
OLH1327	TTTCTTTTTTTTAGTAATTTTTCTTTAGATTTATCGGAAT ACTTAGAATTCGAGCTCGTTTAAAC
OLH1328	TTTCTTTTTTTTAGTAATTTTTCTTTAGATTTATCGGAAT ACTTA GTTGTAAAACGACGGCCAGT
OLH1329	TCGGTAGATCGAAAACACAGG
OLH1337	TCCACAACCACCTTCATCTTC
OLH1362	TGAAAGTTATGACTAACTTACCAGCAAGGGCGCCTCCA GCATTACAAGGTCCCTTTTC
OLH1363	TAAGTTAGTCATAACTTTCATAGGATC
OLH1416	AGTAGAATTCATGCGTAAAGGTGAAGAACTGTTCAC
OLH1417	CAACAAGCTTTTTGTAGAGTTCATCCATG
OLH1459	CAAGACTAAGCACAACACAAATTTCCAAAGAGGAATTA TCCACAGGAAACAGCTATGACC

OLH1460	GTTTGCCCATGAAGTAAATAAGGACTTTCCATATTTAAAGGTTGTAAAACGACGGCCAGT
OLH1466	GAATTATCTCCCATTACACAAGACGCTCCAACATCATC
OLH1467	GTCTTGTGTAATGGGAGATAATTCCTCTTTG
