# Supplementary material for: The GCKIII Kinase Sps1 and the 14-3-3 Isoforms, Bmh1 and Bmh2, Cooperate to Ensure Proper Sporulation in Saccharomyces cerevisiae
Source: PLoS One. 2014 Nov 19;9(11):e113528. doi: 10.1371/journal.pone.0113528 (PMC4237420; doi:10.1371/journal.pone.0113528)
Supplement: Table S3 — S. cerevisiae strains used in this study. (PDF) [file pone.0113528.s008.pdf]

SUPPORTING TABLE S3: *S. cerevisiae* strains used in this study

Yeast strains

Strain	Genotype	Source
LH177	MAT**a**/MATα *ho::hisG/ho::hisG lys2/lys2 ura3/ura3 leu2/leu2 his3/his3 trp1ΔFA/trp1ΔFA*	[50]
LH791	MAT**a**/MATα *ho::hisG/ho::hisG lys2/lys2 ura3/ura3 leu2/leu2 his3/his3 trp1ΔFA/trp1ΔFA SPS1-zz-URA3*$^{K.l}$*/SPS1-zz-URA3*$^{K.l}$	This Study
LH872	MAT**a**/MATα *ho::hisG/ho::hisG lys2/lys2 ura3/ura3 leu2/leu2 his3/his3 trp1ΔFA/trp1ΔFA sps1::LEU2*$^{C.g.}$*/sps1::LEU2*$^{C.g.}$	This Study
LH875	MAT**a**/MATα *ho::hisG/ho::hisG lys2/lys2 ura3/ura3 leu2/leu2 his3/his3 trp1ΔFA/trp1ΔFA SPS1-13x myc-TRP1/SPS1-13x myc-TRP1*	This Study
LH902	MAT**a**/MATα *ho::hisG/ho::hisG lys2/lys2 ura3/ura3 leu2/leu2 his3/his3 trp1ΔFA/trp1ΔFA HTB2-mCherry-TRP1*$^{C.g.}$*/HTB2-mCherry-TRP1*$^{C.g.}$	[48]
LH951	MAT**a** *ho::hisG lys2 ura3 leu2 his3 trp1ΔFA URA3-SPS1*	This Study
LH952	MAT**a** *ho::hisG lys2 ura3 leu2 his3 trp1ΔFA SBP-sps1(S345::URA3)*	This Study
LH953	MAT**a**/MATα *ho::hisG/ho::hisG lys2/lys2 ura3/ura3 leu2/leu2 his3/his3 trp1ΔFA/trp1ΔFA sps1ΔEPG-zz-URA3*$^{K.l}$*/sps1ΔEPG-zz-URA3*$^{K.l}$	This Study
LH954	MAT**a**/MATα *ho::hisG/ho::hisG lys2/lys2 ura3/ura3 leu2/leu2 his3/his3 trp1ΔFA/trp1ΔFA SBP-SPS1/SBP-SPS1*	This Study
LH955	MAT**a**/MATα *ho::hisG/ho::hisG lys2/lys2 ura3/ura3 leu2/leu2 his3/his3 trp1ΔFA/trp1ΔFA SBP-sps1-T12A/SBP-sps1-T12A*	This Study
LH956	MAT**a**/MATα *ho::hisG/ho::hisG lys2/lys2 ura3/ura3 leu2/leu2 his3/his3 trp1ΔFA/trp1ΔFA dit1::TRP1*$^{C.g.}$*/dit1::TRP1*$^{C.g.}$	This Study
LH957	MAT**a**/MATα *ho::hisG/ho::hisG lys2/lys2 ura3/ura3 leu2/leu2 his3/his3 trp1ΔFA/trp1ΔFA BMH1-GFP-TRP1/BMH1-GFP-TRP1*	This Study
LH958	MAT**a**/MATα *ho::hisG/ho::hisG lys2/lys2 ura3/ura3 leu2/leu2 his3/his3 trp1ΔFA/trp1ΔFA BMH2-GFP-TRP1/BMH2-GFP-TRP1*	This Study

LH959	MAT**a**/MATα *ho::hisG/ho::hisG lys2/lys2 ura3/ura3 leu2/leu2 his3/his3 trp1ΔFA/trp1ΔFA bmh2::URA3/bmh2::URA3*	This Study
LH960	MAT**a**/MATα *ho::hisG/ho::hisG lys2/lys2 ura3/ura3 leu2/leu2 his3/his3 trp1ΔFA/trp1ΔFA HTB2-mCherry-TRP1*$^{C.g.}$*/HTB2-mCherry-TRP1*$^{C.g.}$ *SPS1-zz-URA3*$^{K.l}$*/SPS1-zz-URA3*$^{K.l}$	This Study
LH961	MAT**a**/MATα *ho::hisG/ho::hisG lys2/lys2 ura3/ura3 leu2/leu2 his3/his3 trp1ΔFA/trp1ΔFA HTB2-mCherry-TRP1*$^{C.g.}$*/HTB2-mCherry-TRP1*$^{C.g.}$ *sps1ΔEPG-zz-URA3*$^{K.l}$*/sps1ΔEPG-zz-URA3*$^{K.l}$	This Study
LH962	MAT**a**/MATα *ho::hisG/ho::hisG lys2/lys2 ura3/ura3 leu2/leu2 his3/his3 trp1ΔFA/trp1ΔFA HTB2-mCherry-TRP1*$^{C.g.}$*/HTB2-mCherry-TRP1*$^{C.g.}$ *SBP-SPS1/SBP-SPS1*	This Study
LH963	MAT**a**/MATα *ho::hisG/ho::hisG lys2/lys2 ura3/ura3 leu2/leu2 his3/his3 trp1ΔFA/trp1ΔFA HTB2-mCherry-TRP1*$^{C.g.}$*/HTB2-mCherry-TRP1*$^{C.g.}$ *SBP-SPS1/+*	This Study
LH964	MAT**a**/MATα *ho::hisG/ho::hisG lys2/lys2 ura3/ura3 leu2/leu2 his3/his3 trp1ΔFA/trp1ΔFA HTB2-mCherry-TRP1*$^{C.g.}$*/HTB2-mCherry-TRP1*$^{C.g.}$ *SBP-SPS1/sps1::LEU2*	This Study
LH965	MAT**a**/MATα *ho::hisG/ho::hisG lys2/lys2 ura3/ura3 leu2/leu2 his3/his3 trp1ΔFA/trp1ΔFA HTB2-mCherry-TRP1*$^{C.g.}$*/HTB2-mCherry-TRP1*$^{C.g.}$ *sps1::LEU2/+*	This Study
LH966	MAT**a**/MATα *ho::hisG/ho::hisG lys2/lys2 ura3/ura3 leu2/leu2 his3/his3 trp1ΔFA/trp1ΔFA HTB2-mCherry-TRP1*$^{C.g.}$*/HTB2-mCherry-TRP1*$^{C.g.}$ *sps1::LEU2/sps1::LEU2*	This Study
LH967	MAT**a**/MATα *ho::hisG/ho::hisG lys2/lys2 ura3/ura3 leu2/leu2 his3/his3 trp1ΔFA/trp1ΔFA HTB2-mCherry-TRP1*$^{C.g.}$*/HTB2-mCherry-TRP1*$^{C.g.}$ *SBP-sps1-S345A/SBP-sps1-S345A*	This Study
LH968	MAT**a**/MATα *ho::hisG/ho::hisG lys2/lys2 ura3/ura3 leu2/leu2 his3/his3 trp1ΔFA/trp1ΔFA HTB2-mCherry-TRP1*$^{C.g.}$*/HTB2-mCherry-TRP1*$^{C.g.}$ *SBP-sps1-T12A/SBP-sps1-T12A*	This Study
LH969	MAT**a**/MATα *ho::hisG/ho::hisG lys2/lys2 ura3/ura3 leu2/leu2 his3/his3 trp1ΔFA/trp1ΔFA HTB2-mCherry-TRP1*$^{C.g.}$*/HTB2-mCherry-TRP1*$^{C.g.}$ *SBP-sps1-T12A/+*	This Study

LH970	MAT**a**/MATα *ho::hisG/ho::hisG lys2/lys2 ura3/ura3 leu2/leu2 his3/his3 trp1ΔFA/trp1ΔFA HTB2-mCherry-TRP1*$^{C.g.}$*/HTB2-mCherry-TRP1*$^{C.g.}$ *SBP-sps1-T12A/sps1::LEU2*	This Study
LH971	MAT**a**/MATα *ho::hisG/ho::hisG lys2/lys2 ura3/ura3 leu2/leu2 his3/his3 trp1ΔFA/trp1ΔFA HTB2-mCherry-TRP1*$^{C.g.}$*/HTB2-mCherry-TRP1*$^{C.g.}$ *BMH1-GFP-TRP1/BMH1-GFP-TRP1 BMH2-GFP-TRP1/BMH2-GFP-TRP1*	This Study
LH972	MAT**a**/MATα *ho::hisG/ho::hisG lys2/lys2 ura3/ura3 leu2/leu2 his3/his3 trp1ΔFA/trp1ΔFA HTB2-mCherry-TRP1*$^{C.g.}$*/HTB2-mCherry-TRP1*$^{C.g.}$ *BMH1-GFP-TRP1/BMH1-GFP-TRP1*	This Study
LH973	MAT**a**/MATα *ho::hisG/ho::hisG lys2/lys2 ura3/ura3 leu2/leu2 his3/his3 trp1ΔFA/trp1ΔFA HTB2-mCherry-TRP1*$^{C.g.}$*/HTB2-mCherry-TRP1*$^{C.g.}$ *BMH2-GFP-TRP1/BMH2-GFP-TRP1*	This Study
LH974	MAT**a**/MATα *ho::hisG/ho::hisG lys2/lys2 ura3/ura3 leu2/leu2 his3/his3 trp1ΔFA/trp1ΔFA HTB2-mCherry-TRP1*$^{C.g.}$*/HTB2-mCherry-TRP1*$^{C.g.}$ *BMH1-GFP-TRP1/BMH1-GFP-TRP1 sps1::LEU2/sps1::LEU2*	This Study
LH975	MAT**a**/MATα *ho::hisG/ho::hisG lys2/lys2 ura3/ura3 leu2/leu2 his3/his3 trp1ΔFA/trp1ΔFA HTB2-mCherry-TRP1*$^{C.g.}$*/HTB2-mCherry-TRP1*$^{C.g.}$ *BMH2-GFP-TRP1/BMH2-GFP-TRP1 sps1::LEU2/sps1::LEU2*	This Study
LH976	MAT**a**/MATα *ho::hisG/ho::hisG lys2/lys2 ura3/ura3 leu2/leu2 his3/his3 trp1ΔFA/trp1ΔFA HTB2-mCherry-TRP1*$^{C.g.}$*/HTB2-mCherry-TRP1*$^{C.g.}$ *sps1::HIS3/sps1::HIS3*	This Study
LH977	MAT**a**/MATα *ho::hisG/ho::hisG lys2/lys2 ura3/ura3 leu2/leu2 his3/his3 trp1ΔFA/trp1ΔFA HTB2-mCherry-TRP1*$^{C.g.}$*/HTB2-mCherry-TRP1*$^{C.g.}$ *BMH1-GFP-TRP1/BMH1-GFP-TRP1 bmh2::URA3/bmh2::URA3*	This Study
LH978	MAT**a**/MATα *ho::hisG/ho::hisG lys2/lys2 ura3/ura3 leu2/leu2 his3/his3 trp1ΔFA/trp1ΔFA HTB2-mCherry-TRP1*$^{C.g.}$*/HTB2-mCherry-TRP1*$^{C.g.}$ *BMH2-GFP-TRP1/BMH2-GFP-TRP1 bmh1::TRP1/bmh1::TRP1*	This Study
LH979	MAT**a**/MATα *ho::hisG/ho::hisG lys2/lys2 ura3/ura3 leu2/leu2 his3/his3 trp1ΔFA/trp1ΔFA HTB2-mCherry-TRP1*$^{C.g.}$*/HTB2-mCherry-TRP1*$^{C.g.}$ *bmh1::TRP1/+ bmh2::URA3/+*	This Study
LH980	MAT**a**/MATα *ho::hisG/ho::hisG lys2/lys2 ura3/ura3 leu2/leu2 his3/his3 trp1ΔFA/trp1ΔFA HTB2-mCherry-TRP1*$^{C.g.}$*/HTB2-mCherry-TRP1*$^{C.g.}$ *bmh1::TRP1/bmh1::TRP1*	This Study

LH981	MAT**a**/MATα *ho::hisG/ho::hisG lys2/lys2 ura3/ura3 leu2/leu2 his3/his3 trp1ΔFA/trp1ΔFA HTB2-mCherry-TRP1*$^{C.g.}$*/HTB2-mCherry-TRP1*$^{C.g.}$ *bmh2::URA3/bmh2::URA3*	This Study
LH982	MAT**a**/MATα *ho::hisG/ho::hisG lys2/lys2 ura3/ura3 leu2/leu2 his3/his3 trp1ΔFA/trp1ΔFA HTB2-mCherry-TRP1*$^{C.g.}$*/HTB2-mCherry-TRP1*$^{C.g.}$ *bmh1::TRP1/bmh1::TRP1 bmh2::URA3/+*	This Study
LH983	MAT**a**/MATα *ho::hisG/ho::hisG lys2/lys2 ura3/ura3 leu2/leu2 his3/his3 trp1ΔFA/trp1ΔFA HTB2-mCherry-TRP1*$^{C.g.}$*/HTB2-mCherry-TRP1*$^{C.g.}$ *bmh1::TRP1/+ bmh2::URA3/bmh2::URA3*	This Study
LH984	MAT**a**/MATα *ho::hisG/ho::hisG lys2/lys2 ura3/ura3 leu2/leu2 his3/his3 trp1ΔFA/trp1ΔFA HTB2-mCherry-TRP1*$^{C.g.}$*/HTB2-mCherry-TRP1*$^{C.g.}$ *bmh1::TRP1/+ bmh2::URA3/+ sps1::LEU2/+*	This Study
LH985	MAT**a**/MATα *ho::hisG/ho::hisG lys2/lys2 ura3/ura3 leu2/leu2 his3/his3 trp1ΔFA/trp1ΔFA HTB2-mCherry-TRP1*$^{C.g.}$*/HTB2-mCherry-TRP1*$^{C.g.}$ *bmh1::TRP1/+ bmh2::URA3/+ sps1::LEU2/SBP-sps1-T12A*	This Study
LH986	MAT**a**/MATα *ho::hisG/ho::hisG lys2/lys2 ura3/ura3 leu2/leu2 his3/his3 trp1ΔFA/trp1ΔFA HTB2-mCherry-TRP1*$^{C.g.}$*/HTB2-mCherry-TRP1*$^{C.g.}$ *superfolderGFP-SPS1/superfolderGFP-SPS1*	This Study
LH987	MAT**a**/MATα *ho::hisG/ho::hisG lys2/lys2 ura3/ura3 leu2/leu2 his3/his3 trp1ΔFA/trp1ΔFA HTB2-mCherry-TRP1*$^{C.g.}$*/HTB2-mCherry-TRP1*$^{C.g.}$ *superfolderGFP-sps1-T12A/superfolderGFP-sps1-T12A*	This Study
Y5050	MAT**a**/MATα *leu2/leu2 ARG4/arg4-NspI RME1/rme1Δ::LEU2 hoΔ::LYS2/hoΔ::LYS2 ura3/ura3 trp1::hisG/trp1::hisG lys2/lys2 GFP-SPS1/GFP-SPS1*	[11]

Yeast strains with plasmids

Strain	Genotype	Source
LH988	LH872 plus pCS20	This Study
LH989	LH872 plus pCS28	This Study
LH990	LH954 plus pRS424-ssGFP	This Study

LH991	LH955 plus pRS424-ssGFP	This Study
LH992	LH872 plus pCS98	This Study
LH993	LH872 plus pCS96	This Study
LH994	LH872 plus pCS107	This Study
LH995	LH976 plus pCS22	This Study
LH996	LH976 plus pCS65	This Study
LH997	LH976 plus pCS130	This Study
LH998	LH976 plus pCS75	This Study
LH999	LH976 plus pCS60	This Study
LH1000	LH976 plus pCS78	This Study
